# Self-reported insomnia symptoms, sleep duration, chronotype and the risk of acute myocardial infarction (AMI): a prospective study in the UK Biobank and the HUNT Study

**DOI:** 10.1007/s10654-023-00981-x

**Published:** 2023-03-27

**Authors:** Nikhil Arora, Rebecca Claire Richmond, Ben Michael Brumpton, Bjørn Olav Åsvold, Håvard Dalen, Eivind Schjelderup Skarpsno, Linn Beate Strand

**Affiliations:** 1 K.G. Jebsen Center for Genetic Epidemiology, Department of Public Health and Nursing, Norwegian University of Science and Technology, Trondheim, Norway; 2 Population Health Sciences, Bristol Medical School, University of Bristol, Bristol, UK; 3 MRC Integrative Epidemiology Unit, University of Bristol, Bristol, UK; 4 Department of Medicine, St. Olavs Hospital, Trondheim, Norway; 5 HUNT Research Centre, Department of Public Health and Nursing, Norwegian University of Science and Technology, Levanger, Norway; 6 Department of Endocrinology, Clinic of Medicine, St. Olavs Hospital, Trondheim, Norway; 7 Department of Circulation and Medical Imaging, Norwegian University of Science and Technology, Trondheim, Norway; 8 Department of Medicine, Levanger Hospital, Nord-Trøndelag Hospital Trust, Levanger, Norway; 9 Clinic of Cardiology, St. Olavs Hospital, Trondheim, Norway; 10 Department of Public Health and Nursing, Norwegian University of Science and Technology, Trondheim, Norway; 11 Department of Neurology and Clinical Neurophysiology, St. Olavs Hospital, Trondheim, Norway

**Keywords:** Insomnia, Sleep duration, Chronotype, Myocardial infarction, Prospective study

## Abstract

Insomnia and short/long sleep duration increase the risk of AMI, but their interaction with each other or with chronotype is not well known. We investigated the prospective joint associations of any two of these sleep traits on risk of AMI. We included 302 456 and 31 091 participants without past AMI episodes from UK Biobank (UKBB; 2006–10) and the Trøndelag Health Study (HUNT2; 1995–97), respectively. A total of 6 833 and 2 540 incident AMIs were identified during an average 11.7 and 21.0 years follow-up, in UKBB and HUNT2, respectively. Compared to those who reported normal sleep duration (7–8 h) without insomnia symptoms, the Cox proportional hazard ratios (HRs) for incident AMI in UKBB among participants who reported normal, short and long sleep duration with insomnia symptoms were 1.07 (95% CI 0.99, 1.15), 1.16 (95% CI 1.07, 1.25) and 1.40 (95% CI 1.21, 1.63), respectively. The corresponding HRs in HUNT2 were 1.09 (95% CI 0.95, 1.25), 1.17 (95% CI 0.87, 1.58) and 1.02 (95% CI 0.85, 1.23). The HRs for incident AMI in UKBB among evening chronotypes were 1.19 (95% CI 1.10, 1.29) for those who had insomnia symptoms, 1.18 (95% CI 1.08, 1.29) for those with short sleep duration, and 1.21 (95% CI 1.07, 1.37) for those with long sleep duration, compared to morning chronotypes without another sleep symptom. The relative excess risk for incident AMI in UKBB due to interaction between insomnia symptoms and long sleep duration was 0.25 (95% CI 0.01, 0.48). Insomnia symptoms with long sleep duration may contribute more than just an additive effect of these sleep traits on the risk of AMI.

**Supplementary Information:**

The online version contains supplementary material available at 10.1007/s10654-023-00981-x.

## Introduction

Globally, more than 8.9 million people die of coronary heart disease (CHD) each year [1]. Some well-known modifiable factors that increase the risk of CHD incidence are dyslipidaemia, hypertension, obesity, diabetes and cigarette smoking [2]. A substantial proportion of CHD, including acute myocardial infarction (AMI) cannot be explained by these known risk factors, and its global burden makes it important to detect novel risk factors [3]. Sleep plays an important role in maintaining health and well-being [4]. Sleep disorders have been associated with several adverse health conditions, including those related to cardiovascular health such as hypertension [5–7], obesity [8, 9], and dyslipidemia [10].

It is estimated that 33% of the population suffer from one or more insomnia symptoms, i.e. trouble falling asleep, frequent awakenings during night, or too early awakening [11, 12], and its prevalence is increasing [13]. We have previously found in the second wave of the Trøndelag Health Study (HUNT2) that individual insomnia symptom(s) and number of insomnia symptoms are associated with incident AMI [14]. Long and short sleep durations have also been found to be associated with increased risk of incident AMI [15], thus indicating the presence of a U-shaped association [16]. Chronotype referred to an individuals’ preference for sleep timing, where a morning person prefers to get up and go to bed early (early bird), while an evening person prefers to get up and go to bed late (night owl) [17], has also been suggested as potential risk factor for AMI [18]. Only a few studies have investigated this association, and the evidence is not consistent with both morning and evening chronotypes found to be at risk of cardiovascular disease (CVD) [19–21].

Although these sleep traits (insomnia symptoms, sleep duration, and chronotype) are interrelated [22–24], most epidemiological studies have evaluated them as distinct entities without consideration of the others. Insomnia symptoms with objective short sleep duration, suggested to be the most biologically severe insomnia disorder phenotype [25], is associated with higher risk of CVD incidence [26]. Moreover, a study reported higher frequency of insomnia symptoms, and long or short sleep duration among evening than morning chronotypes, suggesting evening chronotypes may be more predisposed to sleep disturbances and/or its related consequences [27]. Despite the availability of self-reported sleep traits from large epidemiological studies and the evidence highlighting the complex nature of coexisting sleep traits phenotypes [22–24], the associations of coexisting sleep traits on incident AMI are not well explored.

Given the complex relationship between insomnia symptoms, sleep duration and chronotype and the scarce amount of research on the joint associations of these risk factors on AMI, we prospectively investigated the joint associations of any two self-reported sleep traits together (i.e., insomnia symptoms and sleep duration; insomnia symptoms and chronotype; and chronotype and sleep duration) on subsequent risk of incident AMI in two large population-based cohorts – the UK Biobank and the HUNT2. We also investigated the associations of these sleep traits individually on the risk of incident AMI in the same study samples.

## Methods

### Study population

#### UK Biobank (UKBB)

UKBB is a population-based prospective study of middle-aged adults (ranging from 40 to 69 years) recruited during March 2006–July 2010 and living within 25 miles of one of the 22 study assessment centres located throughout England, Scotland and Wales.

More than 9.2 million individuals were invited and 502 460 participated. The participants signed an electronic consent and completed a touchscreen questionnaire along with a brief computer-assisted interview. They provided detailed information about their lifestyle, physical measures and had blood, urine and saliva samples collected and stored for future analysis, as described elsewhere [28].

UKBB received ethical approval from the National Health Service (NHS) Research Ethics Service (reference number 11/NW/0382). The UKBB database was formed in accordance with the Declaration of Helsinki.

#### HUNT2

All inhabitants aged 20 years or older were invited to participate during a four-phase population-based health survey (the HUNT Study) in the Trøndelag County of Norway, first in 1984–86 (HUNT1), then in 1995–97 (HUNT2), and 2006–08 (HUNT3), and last in 2017–19 (HUNT4). This study is based on data from HUNT2.

In total, 94 187 individuals were invited during 1995–97 and 65 228 (69.3%) participated [29]. The invitation letter was sent by mail along with a self-administered questionnaire. The participants attended examination stations where clinical examination was performed, and blood samples were drawn by trained personnel. A second questionnaire was handed out at the examination site. Detailed information regarding the HUNT2 study has been published elsewhere [30].

The HUNT Study was approved by the Data Inspectorate of Norway and recommended by the Regional Committee for Ethics in Medical Research (REK; reference number 152/95/AH/JGE). The ethical approval for conducting this study was also obtained from the Regional Committee for Ethics in Medical Research (REK nord; reference number 2020/47206). The HUNT Study was conducted in accordance with the Declaration of Helsinki.

### Exposures

#### Insomnia symptoms

In both UKBB and HUNT2, insomnia symptoms were defined as difficulty falling asleep, difficulty maintaining sleep or waking up too early without any related daytime impairment. Thus, our definition of insomnia is not aligned with established frameworks for classification of insomnia [31].

In the UKBB, the participants were asked: “Do you have trouble falling asleep at night or do you wake up in the middle of the night?” with response options “Never/rarely”, “Sometimes”, “Usually” or “Prefer not to answer”, and 500 956 participants (99.7%) answered this insomnia symptoms question. Participants were classified as having insomnia symptoms if they answered “Usually”, otherwise they were classified as having no insomnia symptoms.

In HUNT2, insomnia symptoms were assessed by the following two questions: “Have you had difficulty falling asleep in the last month?” and “During the last month, have you woken too early and not been able to get back to sleep?” with response options “Never”, “Sometimes”, “Often” or “Almost every night”, and 54 322 participants (83.3%) answered one or both of these insomnia symptom questions. Participants who responded “Often” or “Almost every night” to at least one of these questions were classified as having insomnia symptoms. For the participants in HUNT2 answering only one of the insomnia symptom questions, we did the following: (1) if they answered “Often” or “Almost every night” to one of the questions, but did not answer the other, they were classified as having insomnia symptoms, and (2) if they answered “Never” or “Sometimes” to one of the questions, but did not answer the other, they were excluded to avoid possible misclassification.

#### Sleep duration

In UKBB, participants were asked about sleep duration the last four weeks: “About how many hours sleep do you get in every 24 h? (please include naps)”. In HUNT2, participants were asked about sleep duration: “How many hours do you usually spend lying down (i.e. sleeping and/or napping) during a 24-h period?”. In UKBB and HUNT2, 498 245 (99.2%) and 53 203 (81.6%) participants reported their sleep duration, respectively. A three-level categorical variable was created defining sleep duration as “Short” (6 h or less), “Normal” (7–8 h) or “Long” (9 h or more) as per recommendations from the National Sleep Foundation [32]. Extreme responses of less than 3 h or more than 18 h were excluded to avoid improbable short or long sleep durations confounded by poor health.

#### Chronotype

In UKBB, 496 281 (98.8%) participants reported chronotype (“Do you consider yourself to be?” with response options “Definitely a ‘morning’ person”, “More a ‘morning’ than ‘evening’ person”, “More an ‘evening’ than a ‘morning’ person”, “Definitely an ‘evening’ person”, “Do not know”, or “Prefer not to answer”). Chronotype was not reported in HUNT2. Chronotype was dichotomized categorizing the alternatives “Definitely a ‘morning’ person” or “More a ‘morning’ than ‘evening’ person” as “Morning” chronotype; and “More an ‘evening’ than a ‘morning’ person” or “Definitely an ‘evening’ person” as “Evening” chronotype. The alternatives “Do not know” or “Prefer not to answer” were excluded.

### Outcome ascertainment

In UKBB, follow-up for AMI incidence was conducted via linkage to the Hospital Episode Statistics (HES) for England, Scottish Morbidity Record (SMR) and Patient Episode Database for Wales (PEDW) where health-related outcomes had been defined by International Classification of Diseases (ICD)-9 and ICD-10 codes (Field IDs: 41270, 41271, 41280 and 41281). Mortality information was obtained from NHS Digital for participants in England and Wales and from the NHS Central Register (part of the National Records of Scotland) for participants in Scotland where cause of death had been defined by ICD-10 codes (Field IDs: 40001 and 40000).

In HUNT2, the participants were followed up for incident AMI, either identified at hospitals or by the National Cause of Death Registry. Hospitalizations for AMI were identified through a linkage with medical records from the three hospitals (St. Olavs Hospital, Levanger Hospital and Namsos Hospital) of Trøndelag County in which health-related outcomes had been defined by ICD-9 and ICD-10 codes. Death records were identified by a linkage with the National Cause of Death Registry where cause of death had been defined by ICD-10 codes.

**Fig. 1:**
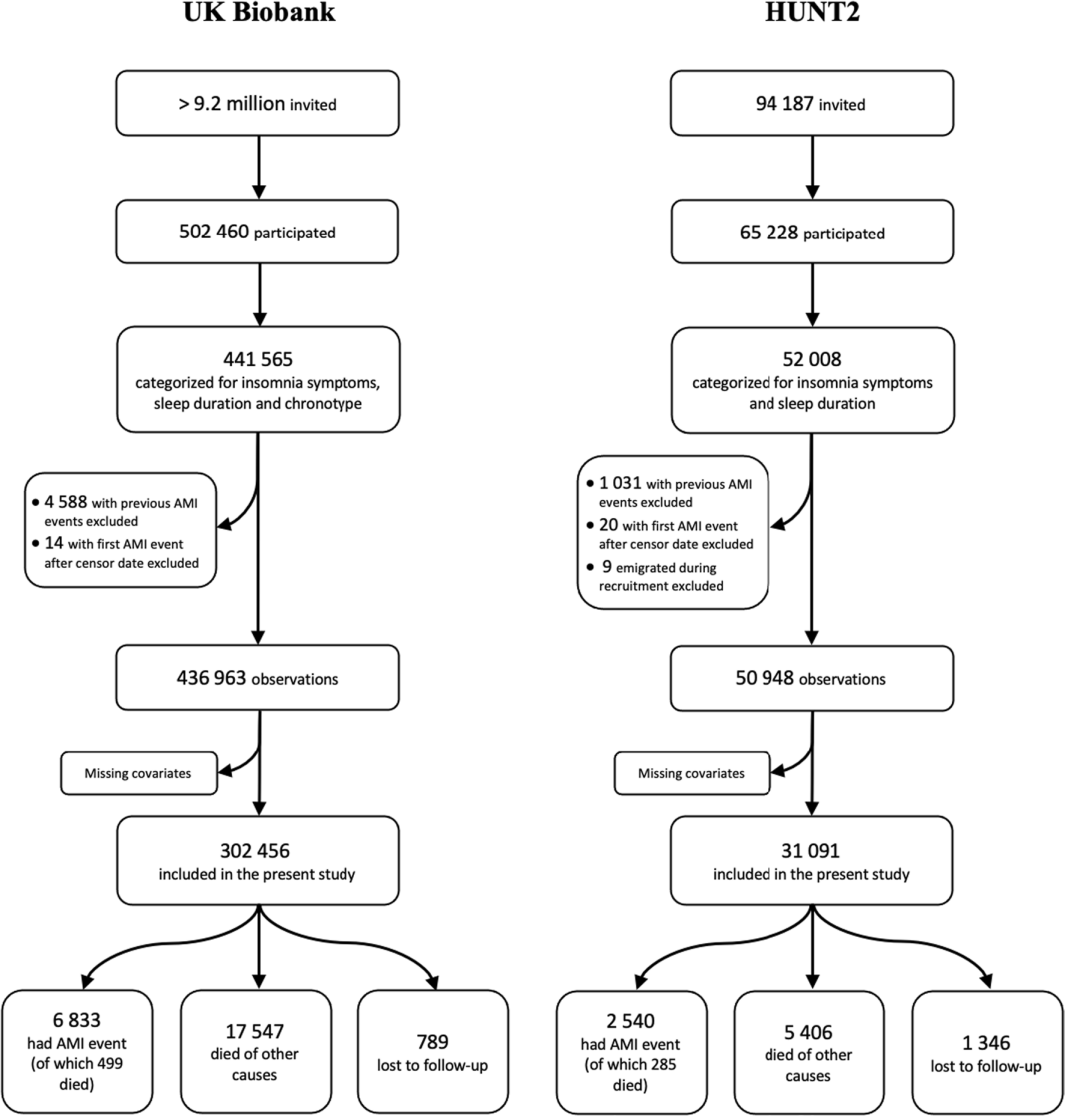
Flow chart of the participant selection process

Incident AMI was defined as the first occurrence of either hospitalization or death due to AMI between date of participation and end of follow-up. All participants with any episode(s) of AMI before their date of participation were excluded. We used ICD-9 code 410 and ICD-10 codes I21 and I22 to identify hospitalizations and deaths due to AMI before and after baseline. A flow chart of participant selection process for our analyses is summarized in Fig. 1.

### Covariates

Information on socio-demographic (i.e. age, gender, marital status, ethnicity (for UKBB only), education and employment status) and lifestyle factors (i.e. smoking, alcohol intake, shift work, physical activity and use of sleep medication(s)) was collected by means of a self-administered questionnaire. A clinical examination was conducted by trained staff where measurements on weight, height, and blood pressure were recorded and blood samples were collected.

For UKBB, marital status was categorized as “Married” or “Unmarried”, while for HUNT2 it was categorized as “Married”, “Unmarried” or “Separated/Divorced/Widowed”. The information on alcohol intake frequency was categorized for “Never”, “Monthly”, “Weekly” or “Daily” alcohol intake. Smoking status of the participants were categorized as “Never”, “Previous” or “Current” smoker. Body mass index (BMI) was computed by dividing weight (in kgs) by the squared value of height (in metres) and was analysed as a continuous variable. The information on physical activity was categorized as “Low/inactive”, “Moderate” or “High” level of physical activity based on International Physical Activity Questionnaire (IPAQ) grouping. Education attainment was categorized as “Primary” (10 years or less), “Secondary” (11–13 years) or “Tertiary” (14 years or more) level of education. Information on shift work/night shifts was analysed as a dichotomous “Yes” or “No” variable. Employment status was categorized as “Employed” or “Not employed”. For UKBB, ethnic background was categorized as “White”, “Mixed”, “Asian/Asian British”, “Black/Black British”, “Chinese” or “Other” ethnic groups, to account for ethnic heterogeneity. We did not adjust for ethnicity in HUNT2, since the population of Nord-Trøndelag is mostly white (less than 3% non-Caucasians) [30]. For UKBB, the Townsend deprivation index (TDI) was used as a continuous variable to account for varying socioeconomic disparities and urban-rural mix within the UK. In HUNT2, education attainment was used to capture any socioeconomic differences. Systolic blood pressure, blood cholesterol levels and blood glucose levels were collected on clinical and laboratory examination and were analysed as continuous variables. Fasting time for UKBB and time since last meal for HUNT2 were used as a continuous variable. Use of sleep medication(s) was ascertained from self-reported use of medications and was analysed as a dichotomous “Yes” or “No” variable. For UKBB, depression and anxiety were categorized as “Yes” or “No” based on diagnosis mapped as ICD-10 codes until the summer of 2019 based on hospital, primary care or self-reported health records, while the Hospital Anxiety and Depression score (HADS) was used on an ordinal scale for HUNT2.

Further details on how the covariates were handled are provided in the supplementary material.

### Statistical analysis

We analysed UKBB and HUNT2 separately. We used Cox proportional hazard models to examine the prospective associations of self-reported insomnia symptoms, sleep duration and chronotype individually on the subsequent risk of incident AMI. We then assessed the joint associations of two sleep traits together i.e., insomnia symptoms and sleep duration; insomnia symptoms and chronotype; and chronotype and sleep duration on the risk of incident AMI. Each participant was followed until either first incident AMI, death, loss to follow-up or until end of follow-up (March 23, 2021 for UKBB and December 31, 2020 for HUNT2). We calculated the number of incident AMI events, person-years at risk and hazard ratios (HRs) with 95% confidence intervals (CIs) using different models adjusting for potential confounding factors.

We reviewed the literature and created Directed Acyclic Graphs (DAGs) to select covariates that could cause both sleep disturbances and AMI. First, we adjusted for age and gender (Model 1). In our main model (Model 2), we further adjusted for marital status, alcohol intake, smoking status, BMI, physical activity, education, shift work/night shifts and employment status. We also adjusted for TDI and ethnicity in Model 2 for our analyses on UKBB. Lastly in Model 3, we additionally included systolic blood pressure, blood cholesterol levels, blood glucose levels, use of sleep medication(s), depression, and anxiety which may be both confounders and/or mediators for the associations under study. Because blood samples were non-fasting, blood laboratory investigations especially for cholesterol and glucose levels could be influenced by time between last meal and venepuncture, so we also adjusted for time since last meal in Model 3.

We tested the proportionality of hazards using log-log curves and Schoenfeld residuals test. The joint associations of any two sleep traits together on the subsequent risk of incident AMI were assessed by using the relative excess risk due to interaction (RERI) with 95% CIs [33]. In brief, RERI > 0 and the lower limit of 95% CI > 0 suggests a synergistic effect of two sleep traits together on incident AMI, i.e., their joint effect on incident AMI is even greater than the sum of their individual effects [34].

We did formal tests for interaction between each sleep trait and their combinations with age (above and below 65 years) and gender. After reviewer comments, we also examined interaction due to shift work, depression (HADS-Depression score above and below 8 in HUNT2) and anxiety (HADS-Anxiety score above and below 8 in HUNT2). In addition, analyses stratified by age, gender, shift work, depression and anxiety were conducted.

We performed several sensitivity analyses to assess the robustness of our findings. To reduce the possibility of reverse causality as an explanation for the observed associations, we repeated the analyses after excluding the first two years of follow-up. In another sensitivity analyses, we adjusted for any self-reported chronic disorders in Models 2 and 3, as sleep disturbances co-exist with certain illnesses and chronic pain [35]. We repeated the same analyses within UKBB restricting only to the White British sample. To compare the findings from UKBB and HUNT2 with the same study follow-up duration, we repeated the analyses in HUNT2 with end of follow-up until December 31, 2008.

The statistical analyses were conducted using R version 4.1.1 for Mac OS (R Foundation for Statistical Computing, Vienna, Austria).

## Results

Baseline characteristics of the study population according to insomnia symptoms status are displayed in Table 1. The baseline characteristics according to sleep duration and chronotype are displayed in Tables S1 and S2, respectively. In UKBB and HUNT2, the prevalence of insomnia symptoms was 27.3% and 12.5%, respectively. The mean (SD) hours of sleep duration for UKBB and HUNT2 were 7.17 (1.07) hours and 7.94 (1.17) hours, respectively. The prevalence of short sleep duration and long sleep duration was 23.9% and 7.4%, respectively in UKBB; and 6.2% and 23.7%, respectively in HUNT2. The prevalence of evening chronotype in UKBB was 37.2%. Participants who reported insomnia symptoms, long sleep duration or morning chronotype were older and were more likely to be women than men. In HUNT2, males were more likely to sleep for short duration compared to females. Participants who reported insomnia symptoms or long sleep duration were more likely unemployed. In HUNT2, the use of sleep medication(s) was more common among participants who reported insomnia symptoms or long sleep duration. In both cohorts, depression or anxiety was more frequent among participants who reported insomnia symptoms.

**Table 1:** Baseline characteristics of participants from UK Biobank and HUNT2 according to self-reported insomnia symptoms

	**UK Biobank**		**HUNT2**	
	Insomnia symptoms (n = 302 456)		Insomnia symptoms (n = 31 091)	
	No	Yes	No	Yes
**Total, % (n)**	72.7 (219 784)	27.3 (82 672)	87.5 (27 196)	12.5 (3 895)
**Variables, % (n)**				
Male	49.2 (108 116)	39.6 (32 777)	47.7 (12 982)	39.6 (1 542)
Married	74.5 (163 730)	71.0 (58 717)	61.2 (16 634)	61.2 (2 385)
Weekly alcohol intake	50.9 (111 836)	46.7 (38 612)	26.6 (7 235)	25.6 (996)
Current smokers	10.0 (21 965)	11.0 (9 051)	28.3 (7 706)	34.8 (1 354)
Highly physically active	41.2 (90 468)	38.5 (31 836)	38.6 (10 513)	28.9 (1 126)
Tertiary education	48.6 (106 732)	42.7 (35 291)	25.7 (6 984)	18.6 (725)
Shift workers	5.6 (12 261)	4.9 (4 015)	18.5 (5 042)	15.7 (612)
Employed	62.2 (136 694)	52.8 (43 635)	74.2 (20 183)	57.0 (2 222)
Use of sleep medication(s)	0.5 (1 036)	2.0 (1 654)	3.2 (872)	24.9 (970)
Suffering from depression	9.7 (21 210)	16.4 (13 572)	–	–
Suffering from anxiety	5.3 (11 651)	8.8 (7 282)	–	–
**Variables, mean (SD)**				
Age, *years*	55.87 (8.22)	57.25 (7.70)	45.10 (15.04)	51.52 (15.82)
TDI	−1.49 (2.97)	−1.29 (3.09)	–	–
BMI, kg/m^2^	27.14 (4.54)	27.68 (5.01)	26.08 (3.94)	26.47 (4.32)
SBP, mmHg	137.5 (18.61)	137.8 (18.43)	134.10 (19.47)	136.70 (21.15)
Time since last meal, h	3.76 (2.37)	3.81 (2.48)	2.14 (1.91)	2.22 (1.95)
Serum cholesterol, mmol/L	5.70 (1.12)	5.74 (1.16)	5.72 (1.21)	6.00 (1.24)
Blood glucose, mmol/L	5.08 (1.16)	5.16 (1.31)	5.32 (1.31)	5.45 (1.42)
HADS-D scores	–	–	2.92 (2.67)	5.12 (3.65)
HADS-A scores	–	–	3.80 (2.95)	6.51 (4.08)

SD indicates standard deviation; TDI, Townsend deprivation index; BMI, body mass index; SBP, systolic blood pressure; HADS – D scores, Hospital Anxiety and Depression Score – Depression scores; and HADS – A scores, Hospital Anxiety and Depression Score – Anxiety scores

Among 302 456 UKBB participants without previous AMI, a total of 6 833 incident AMIs were observed during a mean (SD) follow-up period of 11.7 (1.9) years. Among 31 091 HUNT2 participants without previous AMI, a total of 2 540 incident AMIs were identified during a mean (SD) follow-up of 21.0 (6.5) years.

### Associations of self-reported individual sleep trait(s) and incident AMI

The age- and gender-adjusted HRs and multivariable adjusted HRs with 95% CIs for incident AMI in relation to self-reported insomnia symptoms, sleep duration and chronotype are presented in Table 2. After adjusting for potential confounders (Model 2), the participants who reported insomnia symptoms had a HR of 1.11 (95% CI 1.05, 1.16) and 1.09 (95% CI 0.98, 1.21) for incident AMI in UKBB and HUNT2, respectively, compared to those without insomnia symptoms. Compared to participants who reported normal sleep duration (7–8 h), the HRs for incident AMI in UKBB were 1.09 (95% CI 1.04, 1.16) and 1.14 (95% CI 1.05, 1.24) for those who reported short sleep duration (6 h or less) and long sleep duration (9 h or more), respectively. The corresponding HRs in HUNT2 were similar for those who reported short sleep duration (HR 1.05; 95% CI 0.89, 1.24), but not for those who reported long sleep duration (HR 0.97; 95% CI 0.88, 1.06). Compared to morning chronotypes, the HR for incident AMI was 1.08 (95% CI 1.03, 1.13) for evening chronotypes in UKBB.

**Table 2:** Hazard ratios (95% confidence intervals) for acute myocardial infarction (AMI) according to self-reported insomnia symptoms, sleep duration and chronotype in UK Biobank (UKBB) and HUNT2

		**Insomnia symptoms**		**Sleep duration**			**Chronotype**	
		No	Yes	Short	Normal	Long	Morning	Evening
**UK Biobank** (n = 302 456)	AMI events/ Person-years	4 784/ 2 583 503	2 049/ 964 868	1 794/ 844 021	4 365/ 2 445 890	674/ 258 460	4 206/ 2 229 937	2 627/ 1 318 434
	Model 1	Ref.	1.19 (1.13, 1.25)	1.20 (1.14, 1.27)	Ref.	1.29 (1.19, 1.40)	Ref.	1.14 (1.08, 1.19)
	Model 2	Ref.	1.11 (1.05, 1.16)	1.09 (1.04, 1.16)	Ref.	1.14 (1.05, 1.24)	Ref.	1.08 (1.03, 1.13)
	Model 3	Ref.	1.08 (1.03, 1.14)	1.09 (1.03, 1.15)	Ref.	1.10 (1.01, 1.19)	Ref.	1.06 (1.01, 1.12)
**HUNT2** (n = 31 091)	AMI events/ Person-years	2 120/ 577 219	420/ 76 391	151/ 41 306	1 668/ 470 344	721/ 141 960	–	–
	Model 1	Ref.	1.17 (1.06, 1.30)	1.15 (0.97, 1.35)	Ref.	1.05 (0.96, 1.15)	–	–
	Model 2	Ref.	1.09 (0.98, 1.21)	1.05 (0.89, 1.24)	Ref.	0.97 (0.88, 1.06)	–	–
	Model 3	Ref.	1.08 (0.96, 1.21)	1.09 (0.93, 1.29)	Ref.	0.94 (0.85, 1.03)	–	–

Model 1, adjusted for age and gender

Model 2, adjusted for covariates in Model 1, along with marital status, alcohol intake frequency, smoking status, body mass index, physical activity, education, Townsend deprivation index (for UKBB), ethnicity (for UKBB), shift work, and employment status

Model 3, adjusted for covariates in Model 2, along with systolic blood pressure, serum cholesterol level, blood glucose level, time since last meal, use of sleep medication(s), depression, and anxiety

### Joint associations of self-reported sleep traits and incident AMI

Table 3 presents HRs with 95% CIs for incident AMI in relation to the joint association of self-reported insomnia symptoms and sleep duration within UKBB and HUNT2. Compared to participants who reported normal sleep duration without insomnia symptoms, the multi-adjusted HR for incident AMI in UKBB was 1.07 (95% CI 0.99, 1.15) for those who reported normal sleep duration with insomnia symptoms, whereas the HR increased to 1.16 (95% CI 1.07, 1.25) for those who reported short sleep duration with insomnia symptoms and 1.40 (95% CI 1.21, 1.63) for those who reported long sleep duration with insomnia symptoms. The corresponding HRs in HUNT2 were similar for those who reported normal sleep duration with insomnia symptoms (HR 1.09; 95% CI 0.95, 1.25), and who reported short sleep duration with insomnia symptoms (HR 1.17; 95% CI 0.87, 1.58), but not for those who reported long sleep duration with insomnia symptoms (HR 1.02; 95% CI 0.85, 1.23). In UKBB, we found statistical evidence for biological interaction beyond additivity for long sleep duration with insomnia symptoms (relative excess risk due to interaction (RERI) 0.25; 95% CI 0.01, 0.48), but no such evidence for short sleep duration with insomnia symptoms (RERI 0.02; 95% CI -0.11, 0.15). In HUNT2, we did not find evidence of interaction beyond additivity for short sleep duration (RERI 0.06; 95% CI -0.36, 0.48) or long sleep duration (RERI -0.04; 95% CI -0.28, 0.20) with insomnia symptoms.

**Table 3:** Hazard ratios (95% confidence intervals) for acute myocardial infarction (AMI) according to the joint association of self-reported insomnia symptoms and sleep duration in UK Biobank (UKBB) and HUNT2.

		**No insomnia symptoms**			**Insomnia symptoms**		
		**Sleep duration**			**Sleep duration**		
		Short	Normal	Long	Short	Normal	Long
**UK Biobank** (n = 302 456)	AMI events/ Person-years	900/ 427 642	3 395/ 1 951 096	489/ 204 765	894/ 416 379	970/ 494 794	185/ 53 695
	Model 1	1.16 (1.08, 1.25)	Ref.	1.22 (1.11, 1.34)	1.32 (1.23, 1.42)	1.12 (1.05, 1.21)	1.72 (1.49, 2.00)
	Model 2	1.07 (0.99, 1.15)	Ref.	1.09 (0.99, 1.20)	1.16 (1.07, 1.25)	1.07 (0.99, 1.15)	1.40 (1.21, 1.63)
	Model 3	1.08 (1.00, 1.16)	Ref.	1.05 (0.96, 1.16)	1.13 (1.04, 1.21)	1.05 (0.98, 1.13)	1.32 (1.14, 1.54)
**HUNT2** (n = 31 091)	AMI events/ Person-years	106/ 32 967	1 420/ 420 985	594/ 123 267	45/ 8 339	248/ 49 360	127/ 18 692
	Model 1	1.10 (0.90, 1.34)	Ref.	1.06 (0.96, 1.17)	1.38 (1.03, 1.86)	1.17 (1.03, 1.34)	1.19 (0.99, 1.43)
	Model 2	1.02 (0.84, 1.24)	Ref.	0.97 (0.88, 1.07)	1.17 (0.87, 1.58)	1.09 (0.95, 1.25)	1.02 (0.85, 1.23)
	Model 3	1.08 (0.89, 1.32)	Ref.	0.94 (0.85, 1.04)	1.18 (0.87, 1.60)	1.08 (0.94, 1.25)	0.97 (0.80, 1.19)

Model 1, adjusted for age and gender

Model 2, adjusted for covariates in Model 1, along with marital status, alcohol intake frequency, smoking status, body mass index, physical activity, education, Townsend deprivation index (for UKBB), ethnicity (for UKBB), shift work, and employment status

Model 3, adjusted for covariates in Model 2, along with systolic blood pressure, serum cholesterol level, blood glucose level, time since last meal, use of sleep medication(s), depression, and anxiety

HRs with 95% CIs for incident AMI in relation to the joint association of self-reported insomnia symptoms and chronotype within UKBB are presented in Table 4. Compared to morning chronotypes without insomnia symptoms, the HRs for incident AMI were 1.08 (95% CI 1.02, 1.15) for evening chronotypes without insomnia symptoms, and 1.11 (95% CI 1.04, 1.18) for morning chronotypes with insomnia symptoms, whereas the HR increased to 1.19 (95% CI 1.10, 1.29) for evening chronotypes with insomnia symptoms. There was no evidence of interaction beyond additivity for evening chronotype with insomnia symptoms (RERI -0.01; 95% CI -0.12, 0.12).

**Table 4:** Hazard ratios (95% confidence intervals) for acute myocardial infarction (AMI) according to the joint association of self-reported insomnia symptoms and chronotype in UK Biobank

		**No insomnia symptoms**		**Insomnia symptoms**	
		**Chronotype**		**Chronotype**	
		Morning	Evening	Morning	Evening
**UK Biobank** (n = 302 456)	AMI events/ Person-years	2 953/ 1 625 404	1 831/ 958 099	1 253/ 604 533	796/ 360 335
	Model 1	Ref.	1.12 (1.06, 1.19)	1.17 (1.10, 1.25)	1.36 (1.26, 1.47)
	Model 2	Ref.	1.08 (1.02, 1.15)	1.11 (1.04, 1.18)	1.19 (1.10, 1.29)
	Model 3	Ref.	1.07 (1.01, 1.14)	1.09 (1.02, 1.17)	1.14 (1.06, 1.24)

Model 1, adjusted for age and gender

Model 2, adjusted for covariates in Model 1, along with marital status, alcohol intake frequency, smoking status, body mass index, physical activity, education, Townsend deprivation index, ethnicity, shift work, and employment status

Model 3, adjusted for covariates in Model 2, along with systolic blood pressure, serum cholesterol level, blood glucose level, time since last meal, use of sleep medication(s), depression, and anxiety

Table 5 presents HRs with 95% CIs for incident AMI in relation to the joint association of self-reported chronotype and sleep duration within UKBB. Compared to participants who reported normal sleep duration with morning chronotype, the HR for incident AMI was 1.08 (95% CI 1.02, 1.15) for those who reported normal sleep duration with evening chronotype, whereas the HR increased to 1.18 (95% CI 1.08, 1.29) for those who reported short sleep duration with evening chronotype and 1.21 (95% CI 1.07, 1.37) for those who reported long sleep duration with evening chronotype. There was no evidence of interaction beyond additivity for short sleep duration (RERI -0.01; 95% CI -0.14, 0.12) or long sleep duration (RERI -0.02; 95% CI -0.21, 0.18) with evening chronotype.

**Table 5:** Hazard ratios (95% confidence intervals) for acute myocardial infarction (AMI) according to the joint association of self-reported chronotype and sleep duration in UK Biobank

		**Morning chronotype**			**Evening chronotype**		
		**Sleep duration**			**Sleep duration**		
		Short	Normal	Long	Short	Normal	Long
**UK Biobank** (n = 302 456)	AMI events/ Person-years	1 141/ 539 398	2 689/ 1 540 774	376/ 149 766	653/ 304 623	1 676/ 905 117	298/ 108 694
	Model 1	1.21 (1.12, 1.29)	Ref.	1.28 (1.15, 1.42)	1.37 (1.26, 1.49)	1.13 (1.06, 1.20)	1.46 (1.29, 1.64)
	Model 2	1.10 (1.03, 1.18)	Ref.	1.14 (1.03, 1.27)	1.18 (1.08, 1.29)	1.08 (1.02, 1.15)	1.21 (1.07, 1.37)
	Model 3	1.09 (1.02, 1.17)	Ref.	1.12 (1.00, 1.24)	1.16 (1.06, 1.27)	1.07 (1.01, 1.14)	1.15 (1.01, 1.29)

Model 1, adjusted for age and gender

Model 2, adjusted for covariates in Model 1, along with marital status, alcohol intake frequency, smoking status, body mass index, physical activity, education, Townsend deprivation index, ethnicity, shift work, and employment status

Model 3, adjusted for covariates in Model 2, along with systolic blood pressure, serum cholesterol level, blood glucose level, time since last meal, use of sleep medication(s), depression, and anxiety

We found no strong statistical evidence of interaction by age for any individual sleep traits in both cohorts (Table S3). However, for the combination of insomnia and chronotype, we found that young or middle-aged adults (< 65 years), who were evening chronotypes without insomnia symptoms or morning chronotypes with insomnia symptoms had an increased risk of AMI compared to morning chronotypes without insomnia symptoms. We did not find the same increased risk of AMI in these phenotypes among the older participants (≥ 65 years). Additionally, we found no statistical evidence of interaction by gender, shift work, depression or anxiety (Tables S4–S7).

### Sensitivity analyses

When excluding the first two years of follow-up, a total of 6 089 and 2 390 AMI events were reported within UKBB and HUNT2, respectively, and the estimated associations remained fairly unchanged, but were less precise (Tables S8–S11). When adjusting for the presence of any chronic disorders, the effect estimates remained essentially unchanged, but were less precise (Tables S12–S15). When restricting the analyses to “White British” participants in UKBB (n = 269 375), similar findings were reported (Tables S16–S19). A total of 1 144 AMI events were reported in HUNT2 until December 31, 2008 (i.e., the mean (SD) follow-up period of 11.6 (2.5) years), and the estimated associations remained fairly unchanged, but were less precise compared to the complete follow-up period of 21.0 years (Tables S20–S21).

## Discussion

This is a population-based study of self-reported insomnia symptoms, sleep duration, and chronotype involving two large European cohorts. In UKBB, we found that those who had insomnia symptoms, short/long sleep duration, or evening chronotype had an increased risk for incident AMI, compared to those who had no insomnia symptoms, normal sleep duration or morning chronotype, respectively. Although participants with the combinations of two sleep traits (i.e., insomnia symptoms, sleep duration, and chronotype) had the greatest risk of incident AMI in UKBB, we found a synergistic association only for insomnia symptoms with long sleep duration. In HUNT2, we observed similar trends for incident AMI among those who had insomnia symptoms, short sleep duration or their combination, with less precise effect estimates possibly due to lack of power. We found no evidence of synergistic association due to the interaction between these sleep traits in HUNT2.

Our findings for insomnia symptoms and risk of incident AMI are in line with that of a prior study on HUNT2 participants with an average of 11.4 years of follow-up, where individual insomnia symptom(s) and cumulative number of insomnia symptoms were associated with increased risk of incident AMI [14]. Compared to this study, we have a longer follow-up for HUNT2 participants (21.0 years) and have combined the insomnia symptoms to match the UKBB definition.

Our findings that short and long sleep duration moderately increased the risk of incident AMI, compared to normal sleep duration in UKBB, are consistent with findings from a prior study on UKBB with median 7.0 years of follow-up [15]. We have a slightly longer follow-up for UKBB participants (11.7 years) and a normal sleep duration reference group (7–8 h instead of 6–9 h) as per the sleep duration recommendations [32]. We found no association between long sleep duration and risk of AMI in HUNT2. These inconsistent findings might be explained by notable differences in the two cohorts. The lower participation rate in UKBB (5.5%) compared to HUNT2 (69.3%) might have caused selection bias. Moreover, the dominance of short sleepers in UKBB and long sleepers in HUNT2 is possibly due to a general time trend towards short sleep duration from 1995–97 (HUNT2) to 2006–10 (UKBB) [36], making the comparison between the two cohorts difficult.

Our findings for evening chronotype and increased risk of incident AMI are consistent with evidence by Fan et al. [18], that followed 4 576 AMI-free participants for a mean of 10.6 years from the Sleep Heart Health Study (SHHS). They reported that participants with sleep onset later than 12 midnight had 62% increased risk of AMI, compared to those with sleep onset between 10:01 PM and 11:00 PM [18]. In our study, we used self-reported information on chronotype that captured not only early/late sleep onset behaviours, but also early/late morning wake-up behaviours which may more accurately depict time of the day when sleep occurs.

Our findings for the joint association of insomnia symptoms with short sleep duration and moderately increased risk of incident AMI are consistent with evidence from a cross-sectional study by Kalmbach et al. involving 3 911 subjects from Evolution of Pathways to Insomnia Cohort (EPIC) study [22]. They found that subjects who had self-reported insomnia disorder with short sleep duration had three times the odds for AMI (Odds ratio 3.23; 95% CI 1.45, 7.21), compared to those who never had insomnia with 6 h or more of sleep duration [22]. Since this was a cross-sectional study, reverse causation is likely as sleep problems are common in patients with CHD [37]. Moreover, they considered only few potential confounders (age, sex and obesity) in their fully-adjusted model. Similarly, a prospective study by Bertisch et al. involving 4 437 CVD-free participants from SHHS followed for a median of 11.4 years, found a 29% increased risk of incident CVD (HR 1.29; 95% CI 1.00, 1.66) for those who had insomnia symptoms with polysomnographic short sleep duration, compared to those who had no insomnia symptoms with 6 h or more of sleep duration [26].

Our findings showing a synergistic association of insomnia symptoms with long sleep duration and increased risk of incident AMI in UKBB are consistent with a prospective study on this phenotype and incident CHD. Sands-Lincoln et al. followed 86 329 postmenopausal women aged 50–79 years from Women’s Health Initiative (WHI) Observational Study for a mean of 10.3 years. They found that women at high risk of insomnia symptoms, defined as WHI Insomnia Rating Scale (WHIIRS) ≥ 9, with 10 h or more sleep duration had 93% increased risk of incident CHD (HR 1.93; 95% CI 1.06, 3.51), compared to those at low risk of insomnia symptoms, defined as WHIIRS < 9, with 7–8 h of sleep duration [23]. Since this study only involved postmenopausal women aged 50–79 years, the reported association of insomnia symptoms with long sleep duration on the risk of CHD may not be generalizable to the general population. Moreover, the observed inconsistencies in the association of this phenotype and incident AMI in HUNT2 and UKBB might be due to possible differences in the two cohorts, as explained above.

To the best of our knowledge, this is the first study to investigate the joint associations of chronotype with insomnia symptoms or short/long sleep duration on the risk of incident AMI and to investigate the statistical evidence for biological interaction beyond additivity due to the conjunct presence of these sleep traits. The conjunct presence of insomnia symptoms with long sleep duration may be a vulnerable phenotype contributing to a greater risk of AMI, than simply an additive effect of insomnia symptoms and long sleep duration.

Sleep debt, which occurs through insomnia and short sleep duration, may result in glucose intolerance, decreased thyrotropin secretion, increased cortisol concentration, increased sympathetic nervous activity [38], and elevated C-reactive protein (CRP) levels [39], which are pathophysiological in the development of hypertension [7], and CVD events [40, 41]. Evening chronotype is associated with abdominal obesity independent of BMI [42], and with altered secretion of adipokines [43], which is directly involved in the pathogenesis of arterial hypertension and an increased cardiometabolic risk [19]. Although evidence on the biological mechanisms involving long sleep duration are limited, the association of long sleep duration on the risk of AMI may be explained by poor sleep quality, depression or other underlying comorbidities [44]. People reporting long sleep duration are more likely to have poor sleep quality due to fragmented sleep with repeated awakenings [44]. Poor sleep quality may also increase sympathetic activity [45] and activate an inflammatory response [46]. The aftermath activation of CRP may inhibit endothelium-dependent vasodilation and nitric oxide synthesis, suggestive to cause arterial stiffness [47] and trigger atherosclerosis [46, 48]. Insomnia symptoms and long sleep duration may have unique and independent biological pathways through which they cause increase in risk of incident AMI, and this could be the cause of their synergistic effect due to interaction.

The strengths of the current study include the use of two large cohorts with information on self-reported insomnia symptoms, sleep duration and chronotype, making it possible to examine the joint association of these traits on the risk of AMI. Incident AMIs were ascertained using linkages of the cohorts through hospital records and death certificates which minimizes the chance of misclassification. Moreover, we had rich information on possible confounders (e.g., sociodemographic, lifestyle, clinical and biochemical factors).

The current study has several limitations. Sleep traits were not assessed objectively using validated measures such as actigraphy or polysomnography, which may have caused some measurement error. It remains therefore uncertain whether self-reported sleep duration in the present study represents time in bed or actual sleep time. However, sleep duration tends to be overestimated by actigraphy [49], and polysomnography is not routinely used for the evaluation of insomnia, because symptoms of trouble falling asleep, frequent awakenings during night, or too early awakenings may not be captured objectively [50]. Insomnia is a highly subjective disorder and is primarily defined by the nature of the complaint, thus relying on medical records could potentially cause misclassification as it is often misreported or not reported in medical records [51]. Furthermore, neither the questionnaire used to collect the sleep complaints was validated in the two cohorts, nor does our definition of insomnia symptoms comply with the established frameworks for classification of insomnia [31]. For instance, we lack information about some night time symptoms (waking up earlier in UKBB and difficulty maintaining sleep in HUNT2), nor did we have information about daytime impairment or if the symptoms occurred at least three times per week for at least 3 months. This may have biased our estimates towards the null as people with clinically diagnosed insomnia may have been misclassified as not having insomnia. Moreover, since the Trøndelag County is located near the Arctic circle, seasonal variations in the amount of daylight could have caused seasonal fluctuations in the sleep habits. However, a prior study on HUNT2 found no evidence of seasonal variation in reports of insomnia symptoms characterized by difficulty falling asleep and maintaining sleep [52]. We did not have information about sleep apnoea or other sleep disorders in our study. However, a European population-based study suggested that the prevalence of other sleep disorders, including obstructive sleep apnoea is only ~ 5% among those who have insomnia symptoms [12]. Also, we adjusted for age, BMI, blood pressure and depression in our analyses, that are some of the strong correlates of both sleep apnoea and CVD [53]. Thus it appears unlikely that sleep apnoea alone could explain the higher risk of AMI among participants with different sleep traits or their combinations in our study. Lastly, our findings from the two cohorts should be carefully compared due to cohort differences as: (1) the low participation rate in UKBB (5.5%) compared to HUNT2 (69.3%) which might have led to selection bias; (2) the self-reported sleep traits were collected more than 10 years apart in the two cohorts; (3) the mean age at baseline were higher in UKBB (56.6 years) than in HUNT2 (48.3 years); and (4) the difference in prevalence of the sleep duration categories in the two cohorts, where short sleepers dominated in UKBB and long sleepers in HUNT2.

## Conclusion

Our study suggests that individual sleep traits i.e., insomnia symptoms, sleep duration and chronotype alone are important phenotypes associated with an increased risk of AMI, and we found evidence of an excess risk due to interaction for insomnia symptoms with long sleep duration. Insomnia symptoms with long sleep duration may be a vulnerable phenotype that needs to be further explored. Thus, subsequent studies investigating sleep problems on the risk of CHD/CVD should consider interaction between insomnia symptoms and long sleep duration, as investigating only one element may provide a partial recognition of clinically relevant sleep phenotype leading to their out of sight health consequences. We would also suggest further studies to apply a Mendelian randomization design using genetic variants as instrument variables for the sleep traits. Such studies could rule out limitations due to residual confounding and reverse causation. Also, studies aimed at exploring potential vascular and metabolic mechanisms behind insomnia, short/long sleep duration and chronotype are warranted to better understand association underlying AMI risk.

## Electronic supplementary material

Below is the link to the electronic supplementary material.

Supplementary Material (DOCX 160 kb)
