# Supplementary material for: Self-reported insomnia symptoms, sleep duration, chronotype and the risk of acute myocardial infarction (AMI): a prospective study in the UK Biobank and the HUNT Study
Source: Eur J Epidemiol. 2023 Mar 27;38(6):643–56. doi: 10.1007/s10654-023-00981-x (PMC10232611; doi:10.1007/s10654-023-00981-x)
Supplement: Supplementary file 1 — Supplementary Material (DOCX 160 kb) [file 10654_2023_981_MOESM1_ESM.docx]

**Supplementary Material**

### Details on handling of covariates

### Clinical information

Information on socio-demographic (i.e. sex, age, marital status, ethnicity (for UKBB only), education and employment status) and lifestyle factors (i.e. smoking, alcohol intake, shift work, physical activity and use of sleep medication(s)) was collected by means of a self-administered questionnaire. A clinical examination was conducted by trained staff and measurements on weight, height, and blood pressure were collected.

In UKBB, the information on marital status was categorized as “Married” for participants who live with their husband/wife/partner and as “Unmarried” for participants not living with their husband/wife/partner. Additionally, the information on numbers living in a household was used to categorize living alone as “Unmarried” for observations having missing information on marital status.

In HUNT2, the information on marital status was categorized into “Unmarried”, “Married” and “Separated/Divorced/Widowed”.

In UKBB, participants were categorized based on their ethnicity as “White”, “Mixed”, “Asian/ Asian British”, “Black/Black British”, “Chinese” or “Other”.

Within UKBB and HUNT2, education was categorized into whether participants had attained “10 years or less” (primary and lower secondary school), “11 - 13 years” (upper secondary school) or “14 years or more” (university/college) of education.

The information on working status from UKBB and HUNT2 was used to create a binary employment status variable with categories “Employed” or “Not employed”.

The information on smoking status was categorized as “Never”, “Previous” or “Current” smoker for UKBB and HUNT2.

In UKBB and HUNT2, the participants were asked about alcohol intake frequency and were categorized as “Never/rarely” for non-drinkers or special occasion drinkers, “Monthly” for those drinking 1 - 3 times a month, “Weekly” for those drinking 1- 4 times a week or “Daily/almost daily” for those drinking even more frequently. In addition, the information concerning alcohol never drinker for HUNT2 was used to categorize never had alcohol as “Never/rarely” for observations having missing information on alcohol intake frequency. Thus, this information will be categorized as “Never/rarely”, “Monthly”, “Weekly” or “Daily/almost daily” alcohol intake.

In UKBB, the participants were asked about doing shift work or working night shifts separately and a proxy variable was made merging these two responses and keeping the highest response category as final. The proxy variable was then dichotomized categorizing the highest categories, i.e. “Usually” or “Always” as “Yes”, and “No” otherwise. In HUNT2, working shifts/at night/on call was dichotomized as “Yes” or “No”. Additionally, the information on current employment/work status from both UKBB and HUNT2 was used to categorize those without having any paid employment or self-employed as “No” for observations having missing information on working shifts/at night/on call.

The physical activity (PA) within UKBB was assessed using adapted questions from the validated short International Physical Activity Questionnaire (IPAQ) [1], following rules published for data processing by IPAQ [2]. IPAQ assessed total physical activity, including walking, moderate, and vigorous PA performed over the last 7 days. The participants were categorized into three mutually exclusive PA categories - “High” (≥ 1h of moderate PA or ≥ ½ h of vigorous PA above basal level of activity on most days), “Moderate” (≥ ½ h of moderate PA above basal level of activity on most days) or “Low/inactive” (anything else) based on a standard scoring criteria [3], where approximately 5000 steps per day was considered as basal activity. In HUNT2, a proxy variable was created based on participants' response to average hours of light and hard PA during leisure time per week in the last year. Light PA was defined as no sweating or not being out of breath, and hard PA as sweating/out of breath. Participants were instructed to include the commute to work as leisure time. We categorized the HUNT2 participants into similar PA categories - “High” defined by ≥ 1h of hard PA regardless of light PA or ≥ 3h of light PA with < 1h of hard PA, “Moderate” defined by ≥ 3h of light PA with no hard PA or < 3h of light PA with < 1h of hard PA, or “Low/inactive” for anything else. A similar categorization strategy for PA was used before by Brumpton *et al* [4]. The reliability and validity of the questions on PA from HUNT2 have been previously reported to be acceptable for hard PA and poor for light PA [5].

In UKBB, the use of sleep medication(s) was ascertained by the self-reported use of medications from the list of sleep medications as used by Daghlas *et al*. [6], along with five other commonly used anxiolytics or sleep medications (list included in Table S18). A dichotomized “Yes” or “No” variable was created for the use of sleep medication(s). In HUNT2, the participants were asked for their use of anxiolytics or sleep medications in the last month and were categorized as “Yes” for daily or weekly intake, and “No” otherwise.

Within UKBB, weight was measured using the Tanita BC-418MA body composition analyser, to the nearest 0.1kg and height was measured using a Seca 202 height measure. Within HUNT2, weight was measured to the nearest 0.5kg and height was measured to nearest 1cm. The participants for UKBB and HUNT2 wore light clothes and no shoes during these measurements. The body mass index (BMI) was computed by dividing weight (in kgs) by the squared value of height (in metres).

In UKBB, blood pressure (both systolic and diastolic) measurements were recorded automatedly (using Omron HEM-705 IT electronic blood pressure monitor) and/or manually (using manual sphygmomanometer). Two sets of measurements were taken at a one-minute interval and the average of two were used in our analyses. The manual readings were only used if automated readings were unavailable. In HUNT2, blood pressure (both systolic and diastolic) measurements were recorded automatedly using a Dinamap 845XT (Critikon) sphygmomanometer based on oscillometry. Three sets of measurements were taken at a one-minute interval and the average of second and third measurements were used in our analyses.

Townsend Deprivation Index (TDI) as a measure for socioeconomic status was used for UKBB. The index was created from census data on housing, employment, car availability and social class based on postal codes of participants, with higher values indicating greater deprivation. Townsend deprivation index has been validated for use in a UK-based population [7].

### Depression and anxiety

For UKBB, hospitals recorded ICD-10 codes - F40 and F41 for anxiety; and F32, F33, F34, F38 and F39 for depression were used to detect participants having anxiety or depression episodes. Two separate binary proxy variables each for anxiety and depression were created using this information categorized as “Yes” or “No”.

For HUNT2, the Hospital Anxiety and Depression Scale (HADS) was used to assess the symptoms of anxiety and depression. The questionnaire consisted of 14 Likert-scaled items (7 for anxiety and 7 for depression) having a four-point scale ranging from 0 (not at all) to 3 (very often). Responses are summed to provide separate scores each for anxiety and depression ranging from 0 to 21. Higher score indicates increased likelihood of anxiety and depression [8]. No somatic items or items regarding sleeping difficulties were included. HADS is a useful tool in the assessment of symptom severity of anxiety and depression both in primary health care and in hospital settings [9]. The psychometric properties of the scale have previously been validated as part of the HUNT study [10].

### Laboratory measurements

For UKBB, a random (non-fasting) blood sample was drawn for each participant at the assessment centres as per standard operating procedure for the UKBB and stored in refrigerators between 2 to 8 °C. Fasting time was recorded as the interval between consumption of food or drink and blood sample(s) being taken. Samples were transferred to a central laboratory for storage and analyses on a daily basis. Serum samples were centrifuged for 10 minutes at 2000 RCF. Serum concentrations of glucose, total cholesterol, HDL cholesterol, and triglycerides were analysed using a Beckman Coulter AU5800 automated analyser. Glucose was measured using hexokinase analysis. Total cholesterol, HDL cholesterol and triglycerides were measured by CHO-POD analysis, enzyme immunoinhibition analysis and GPO-POD analysis, respectively [11].

For HUNT2, a random (non-fasting) blood sample was drawn for each participant and analysed at the Central Laboratory, Levanger Hospital, using a Hitachi 911 Autoanalyzer (Hitachi, Mito, Japan). Serum was separated from blood by centrifugation within 2 hours at the screening site and placed in a refrigerator (4 °C). Time between the last meal and venepuncture was recorded and the samples were sent to the laboratory on the same day or within two to three days (for example on weekends). Serum concentrations of glucose, total cholesterol, HDL cholesterol, and triglycerides were analysed applying reagents from Boehringer Mannheim (Mannheim, Germany). The day-to-day coefficients of variation were 1.3-2.0%, 1.3-1.9%, 2.4%, and 0.7-1.3%, respectively. Glucose was measured using an enzymatic hexokinase method. Total and HDL cholesterol were measured by an enzymatic colorimetric cholesterol esterase method. Measurement of HDL cholesterol was performed after precipitation with phosphor tungsten and magnesium ions. Triglycerides were measured with an enzymatic colorimetric method [12].

**References**

1. Craig CL, Marshall AL, Sjöström M, Bauman AE, Booth ML, Ainsworth BE, et al. International physical activity questionnaire: 12-country reliability and validity. Med Sci Sports Exerc. 2003/08/06 ed. 2003;35:1381–95.

2. International Physical Activity Questionnaire (IPAQ): Guidelines for data processing analysis of the International Physical Activity Questionnaire (IPAQ) - Short and long forms [Internet]. 2005 [cited 2021 Oct 25]. Available from: https://biobank.ndph.ox.ac.uk/showcase/refer.cgi?id=540

3. International Physical Activity Questionnaire (IPAQ): IPAQ scoring protocol [Internet]. 2005 [cited 2021 Oct 25]. Available from: https://sites.google.com/site/theipaq/scoring-protocol

4. Brumpton BM, Langhammer A, Ferreira MAR, Chen Y, Mai X-M. Physical activity and incident asthma in adults: the HUNT Study, Norway. BMJ Open. 2016;6:e013856.

5. Kurtze N, Rangul V, Hustvedt BE, Flanders WD. Reliability and validity of self-reported physical activity in the Nord-Trøndelag Health Study (HUNT 2). Eur J Epidemiol. 2007/03/16 ed. 2007;22:379–87.

6. Daghlas I, Dashti HS, Lane J, Aragam KG, Rutter MK, Saxena R, et al. Sleep Duration and Myocardial Infarction. J Am Coll Cardiol. 2019/09/07 ed. 2019;74:1304–14.

7. Townsend P. Poverty in the United Kingdom: A Survey of Household Resources and Standards of Living. London: Allen Lane and Penguin Books; 1979.

8. Skapinakis P. Hospital Anxiety and Depression Scale (HADS). In: Michalos AC, editor. Encyclopedia of Quality of Life and Well-Being Research [Internet]. Dordrecht: Springer Netherlands; 2014. p. 2930–3. Available from: https://doi.org/10.1007/978-94-007-0753-5_1315

9. Bjelland I, Dahl AA, Haug TT, Neckelmann D. The validity of the Hospital Anxiety and Depression Scale. An updated literature review. J Psychosom Res. 2002/02/08 ed. 2002;52:69–77.

10. Mykletun A, Stordal E, Dahl AA. Hospital Anxiety and Depression (HAD) scale: factor structure, item analyses and internal consistency in a large population. Br J Psychiatry. 2001/12/04 ed. 2001;179:540–4.

11. Elliott P, Peakman TC, UK Biobank. The UK Biobank sample handling and storage protocol for the collection, processing and archiving of human blood and urine. International Journal of Epidemiology. 2008;37:234–44.

12. Holmen J, Midthjell K, Krüger Ø, Langhammer A, Holmen TL, Bratberg GH, et al. The Nord-Trøndelag Health Study 1995–97 (HUNT 2): objectives, contents, methods and participation. Norsk epidemiologi. 2003;13:19–32.

### Supplementary tables

Table S1: Baseline characteristics of participants from UK Biobank and HUNT2 according to self-reported sleep duration.

|  | **UK Biobank** | | |  | **HUNT2** | | |
| --- | --- | --- | --- | --- | --- | --- | --- |
|  | Sleep duration (n = 302 456) | | |  | Sleep duration (n = 31 091) | | |
|  | Short  (≤ 6 hours) | Normal  (7 or 8 hours) | Long  (≥ 9 hours) |  | Short  (≤ 6 hours) | Normal  (7 or 8 hours) | Long  (≥ 9 hours) |
| **Total, % (n)** | 23.9  (72 216) | 68.7  (207 777) | 7.4  (22 463) |  | 6.2  (1 928) | 70.1  (21 792) | 23.7  (7 371) |
| **Variables, % (n)** | | | | | | |  |
| Male | 47.9  (34 598) | 46.4  (96 328) | 44.4  (9 967) |  | 57.9  (1 117) | 47.8  (10 410) | 40.7  (2 997) |
| Married | 68.1  (49 152) | 75.6  (157 045) | 72.3  (16 250) |  | 55.3  (1 067) | 62.9  (13 718) | 57.4  (4 234) |
| Weekly alcohol intake | 47.4  (34 203) | 51.2  (106 383) | 43.9  (9 862) |  | 25.7  (496) | 27.7  (6 047) | 22.9  (1 688) |
| Current smokers | 12.2  (8 841) | 9.4  (19 562) | 11.6  (2 613) |  | 37.8  (729) | 29.1  (6 333) | 27.1  (1 998) |
| Highly physically active | 40.9  (29 502) | 40.6  (84 382) | 37.5  (8 420) |  | 38.7  (746) | 39.4  (8 590) | 31.2  (2 303) |
| Tertiary education | 43.7  (31 534) | 48.8  (101 431) | 40.3  (9 058) |  | 19.1  (368) | 27.0  (5 875) | 19.9  (1 466) |
| Shift workers | 7.6  (5 474) | 4.8  (9 889) | 4.1  (913) |  | 23.0  (444) | 19.0  (4 131) | 14.6  (1 079) |
| Employed | 62.8  (45 343) | 61.2  (127 076) | 35.2  (7 910) |  | 79.0  (1 523) | 78.5  (17 116) | 51.1  (3 766) |
| Use of sleep medication(s) | 1.4  (1 035) | 0.6  (1 290) | 1.6  (365) |  | 5.7  (110) | 4.3  (933) | 10.8  (799) |
| Suffering from depression | 13.3  (9 622) | 10.0  (20 821) | 19.3  (4 339) |  | - | - | - |
| Suffering from anxiety | 7.3  (5 256) | 5.6  (11 640) | 9.1  (2 037) |  | - | - | - |
| **Variables, mean (SD)** | | | | | | |  |
| Age, *years* | 56.11  (7.87) | 56.07  (8.16) | 58.31  (8.07) |  | 42.93  (13.58) | 44.69  (13.86) | 50.28  (18.55) |
| TDI | -1.05  (3.19) | -1.59  (2.91) | -1.21  (3.15) |  | - | - | - |
| BMI, *kg/m^2^* | 27.85  (4.98) | 27.03  (4.51) | 27.93  (5.04) |  | 26.34  (4.10) | 26.03  (3.89) | 26.36  (4.26) |
| SBP, *mmHg* | 137.60  (18.25) | 137.40  (18.59) | 139.10  (19.15) |  | 132.30  (17.75) | 133.40  (18.72) | 138.20  (22.41) |
| Time since last meal, *hours* | 3.94  (2.65) | 3.71  (2.29) | 3.84  (2.52) |  | 2.29  (2.11) | 2.15  (1.91) | 2.13  (1.89) |
| Serum cholesterol, *mmol/L* | 5.70  (1.13) | 5.71  (1.12) | 5.68  (1.21) |  | 5.62  (1.14) | 5.71  (1.19) | 5.92  (1.28) |
| Blood glucose, *mmol/L* | 5.13  (1.26) | 5.08  (1.13) | 5.26  (1.54) |  | 5.26  (1.17) | 5.29  (1.22) | 5.49  (1.63) |
| HADS - D scores | - | - | - |  | 3.66  (3.24) | 3.02  (2.75) | 3.60  (3.17) |
| HADS - A scores | - | - | - |  | 4.84  (3.67) | 4.05  (3.15) | 4.25  (3.38) |

SD indicates standard deviation; TDI, Townsend deprivation index; BMI, body mass index; SBP, systolic blood pressure; HADS – D scores, Hospital Anxiety and Depression Score – Depression scores; and HADS – A scores, Hospital Anxiety and Depression Score – Anxiety scores.

Table S2: Baseline characteristics of participants from UK Biobank according to self-reported chronotype.

|  | **UK Biobank** | |
| --- | --- | --- |
|  | Chronotype (n = 302 456) | |
|  | Morning | Evening |
| **Total, % (n)** | 62.8 (189 978) | 37.2 (112 478) |
| **Variables, % (n)** | | |
| Male | 45.9 (87 231) | 47.7 (53 662) |
| Married | 75.2 (142 816) | 70.8 (79 631) |
| Weekly alcohol intake | 49.7 (94 381) | 49.8 (56 067) |
| Current smokers | 8.1 (15 457) | 13.8 (15 559) |
| Highly physically active | 42.7 (81 205) | 36.5 (41 099) |
| Tertiary education | 46.5 (88 311) | 47.8 (53 712) |
| Shift workers | 4.8 (9 164) | 6.3 (7 112) |
| Employed | 58.5 (111 179) | 61.5 (69 150) |
| Use of sleep medication(s) | 0.8 (1 436) | 1.1 (1 254) |
| Suffering from depression | 10.3 (19 525) | 13.6 (15 257) |
| Suffering from anxiety | 5.8 (11 094) | 7.0 (7 839) |
| **Variables, mean (SD)** | | |
| Age, *years* | 56.79 (7.95) | 55.33 (8.29) |
| TDI | -1.53 (2.96) | -1.28 (3.08) |
| BMI, *kg/m^2^* | 27.20 (4.63) | 27.43 (4.77) |
| SBP, *mmHg* | 138.00 (18.57) | 136.90 (18.52) |
| Time since last meal, *hours* | 3.73 (2.29) | 3.84 (2.58) |
| Serum cholesterol, *mmol/L* | 5.71 (1.13) | 5.71 (1.13) |
| Blood glucose, *mmol/L* | 5.10 (1.17) | 5.11 (1.26) |

SD indicates standard deviation; TDI, Townsend deprivation index; BMI, body mass index; and SBP, systolic blood pressure.

Table S3: Hazard ratios (95% CIs)* for acute myocardial infarction according to insomnia symptoms, sleep duration, chronotype and their joint association stratified by age at 65 years in UK Biobank (UKBB) and HUNT2.

|  | **UK Biobank** | | |  | **HUNT2** | | |
| --- | --- | --- | --- | --- | --- | --- | --- |
|  | **Age < 65 y** | **Age ≥ 65 y** | **P for interaction** |  | **Age < 65 y** | **Age ≥ 65 y** | **P for interaction** |
| **Exposures** |  |  |  |  |  |  |  |
| Insomnia symptoms |  |  |  |  |  |  |  |
| No | Ref. | Ref. | 0.064 |  | Ref. | Ref. | 0.190 |
| Yes | 1.14  (1.07, 1.21) | 1.04  (0.95, 1.14) |  |  | 1.09  (0.95, 1.25) | 1.09  (0.92, 1.29) |  |
|  |  |  |  |  |  |  |  |
| Sleep duration |  |  |  |  |  |  |  |
| Short | 1.11  (1.04, 1.18) | 1.06  (0.95, 1.17) |  |  | 0.97  (0.80, 1.18) | 1.35  (0.96, 1.89) |  |
| Normal | Ref. | Ref. | 0.399 |  | Ref. | Ref. | 0.035 |
| Long | 1.11  (1.00, 1.24) | 1.17  (1.03, 1.32) |  |  | 0.88  (0.78, 1.01) | 1.06  (0.92, 1.21) |  |
|  |  |  |  |  |  |  |  |
| Chronotype |  |  |  |  |  |  |  |
| Morning | Ref. | Ref. | 0.219 |  | - | - |  |
| Evening | 1.10  (1.03, 1.16) | 1.03  (0.94, 1.13) |  |  | - | - |  |
|  |  |  |  |  |  |  |  |
| Insomnia symptoms (INS) and Sleep duration (SLD) |  |  |  |  |  |  |  |
| No INS & Short SLD | 1.07  (0.98, 1.17) | 1.07  (0.92, 1.23) |  |  | 1.01  (0.81, 1.26) | 1.05  (0.66, 1.67) |  |
| No INS & Normal SLD | Ref. | Ref. | 0.390 |  | Ref. | Ref. | 0.014 |
| No INS & Long SLD | 1.06  (0.93, 1.20) | 1.11  (0.96, 1.29) |  |  | 0.87  (0.75, 1.00) | 1.10  (0.94, 1.28) |  |
| INS & Short SLD | 1.20  (1.10, 1.31) | 1.05  (0.92, 1.21) |  |  | 0.90  (0.62, 1.33) | 2.08  (1.29, 3.36) |  |
| INS & Normal SLD | 1.10  (1.00, 1.20) | 1.02  (0.90, 1.15) |  |  | 1.09  (0.92, 1.28) | 1.15  (0.90, 1.46) |  |
| INS & Long SLD | 1.41  (1.16, 1.71) | 1.38  (1.09, 1.74) |  |  | 1.07  (0.81, 1.40) | 1.02  (0.78, 1.32) |  |
|  |  |  |  |  |  |  |  |
| Insomnia symptoms (INS) and Chronotype (CT) |  |  |  |  |  |  |  |
| No INS & Morning CT | Ref. | Ref. | <0.001 |  | - | - |  |
| No INS & Evening CT | 1.15  (1.07, 1.23) | 0.94  (0.85, 1.05) |  |  | - | - |  |
| INS & Morning CT | 1.21  (1.11, 1.31) | 0.94  (0.83, 1.05) |  |  | - | - |  |
| INS & Evening CT | 1.19  (1.08, 1.31) | 1.18  (1.03, 1.35) |  |  | - | - |  |
|  |  |  |  |  |  |  |  |
| Chronotype (CT) and Sleep duration (SLD) |  |  |  |  |  |  |  |
| Morning CT & Short SLD | 1.15  (1.06, 1.25) | 1.00  (0.88, 1.14) |  |  | - | - |  |
| Morning CT & Normal SLD | Ref. | Ref. | 0.171 |  | - | - |  |
| Morning CT & Long SLD | 1.10  (0.95, 1.28) | 1.18  (1.01, 1.38) |  |  | - | - |  |
| Evening CT & Short SLD | 1.18  (1.07, 1.30) | 1.19  (1.00, 1.41) |  |  | - | - |  |
| Evening CT & Normal SLD | 1.13  (1.05, 1.21) | 1.00  (0.89, 1.12) |  |  | - | - |  |
| Evening CT & Long SLD | 1.25  (1.07, 1.46) | 1.14  (0.94, 1.39) |  |  | - | - |  |
|  |  |  |  |  |  |  |  |

CI indicates confidence interval; INS, insomnia symptoms; SLD, sleep duration; and CT, chronotype.

* Adjustments were performed as in Model 2, Table 2.

Table S4: Hazard ratios (95% CIs)* for acute myocardial infarction according to insomnia symptoms, sleep duration, chronotype and their joint association stratified by gender in UK Biobank (UKBB) and HUNT2.

|  | **UK Biobank** | | |  | **HUNT2** | | |
| --- | --- | --- | --- | --- | --- | --- | --- |
|  | **Female** | **Male** | **P for interaction** |  | **Female** | **Male** | **P for interaction** |
| **Exposures** |  |  |  |  |  |  |  |
| Insomnia symptoms |  |  |  |  |  |  |  |
| No | Ref. | Ref. | 0.795 |  | Ref. | Ref. | 0.282 |
| Yes | 1.12  (1.02, 1.23) | 1.10  (1.03, 1.17) |  |  | 1.17  (0.99, 1.37) | 1.03  (0.89, 1.19) |  |
|  |  |  |  |  |  |  |  |
| Sleep duration |  |  |  |  |  |  |  |
| Short | 1.14  (1.02, 1.26) | 1.08  (1.01, 1.15) |  |  | 1.04  (0.76, 1.42) | 1.05  (0.86, 1.28) |  |
| Normal | Ref. | Ref. | 0.676 |  | Ref. | Ref. | 0.801 |
| Long | 1.16  (1.00, 1.36) | 1.13  (1.02, 1.24) |  |  | 0.93  (0.79, 1.08) | 0.99  (0.88, 1.11) |  |
|  |  |  |  |  |  |  |  |
| Chronotype |  |  |  |  |  |  |  |
| Morning | Ref. | Ref. | 0.859 |  | - | - |  |
| Evening | 1.08  (0.99, 1.19) | 1.08  (1.02, 1.14) |  |  | - | - |  |
|  |  |  |  |  |  |  |  |
| Insomnia symptoms (INS) and Sleep duration (SLD) |  |  |  |  |  |  |  |
| No INS & Short SLD | 1.19  (1.01, 1.39) | 1.04  (0.95, 1.13) |  |  | 1.12  (0.77, 1.63) | 0.99  (0.78, 1.25) |  |
| No INS & Normal SLD | Ref. | Ref. | 0.534 |  | Ref. | Ref. | 0.596 |
| No INS & Long SLD | 1.17  (0.98, 1.40) | 1.06  (0.94, 1.18) |  |  | 0.96  (0.80, 1.14) | 0.98  (0.87, 1.11) |  |
| INS & Short SLD | 1.17  (1.03, 1.34) | 1.15  (1.05, 1.27) |  |  | 1.04  (0.61, 1.78) | 1.25  (0.87, 1.78) |  |
| INS & Normal SLD | 1.15  (1.01, 1.30) | 1.04  (0.95, 1.13) |  |  | 1.25  (1.01, 1.54) | 0.98  (0.82, 1.18) |  |
| INS & Long SLD | 1.33  (0.99, 1.79) | 1.43  (1.20, 1.70) |  |  | 1.04  (0.79, 1.35) | 1.02  (0.78, 1.33) |  |
|  |  |  |  |  |  |  |  |
| Insomnia symptoms (INS) and Chronotype (CT) |  |  |  |  |  |  |  |
| No INS & Morning CT | Ref. | Ref. | 0.934 |  | - | - |  |
| No INS & Evening CT | 1.07  (0.95, 1.20) | 1.09  (1.01, 1.16) |  |  | - | - |  |
| INS & Morning CT | 1.10  (0.98, 1.24) | 1.11  (1.03, 1.21) |  |  | - | - |  |
| INS & Evening CT | 1.22  (1.06, 1.40) | 1.17  (1.07, 1.29) |  |  | - | - |  |
|  |  |  |  |  |  |  |  |
| Chronotype (CT) and Sleep duration (SLD) |  |  |  |  |  |  |  |
| Morning CT & Short SLD | 1.12  (0.98, 1.28) | 1.10  (1.01, 1.19) |  |  | - | - |  |
| Morning CT & Normal SLD | Ref. | Ref. | 0.458 |  | - | - |  |
| Morning CT & Long SLD | 1.29  (1.06, 1.57) | 1.09  (0.96, 1.24) |  |  | - | - |  |
| Evening CT & Short SLD | 1.28  (1.09, 1.51) | 1.14  (1.03, 1.26) |  |  | - | - |  |
| Evening CT & Normal SLD | 1.10  (0.98, 1.24) | 1.08  (1.00, 1.16) |  |  | - | - |  |
| Evening CT & Long SLD | 1.10  (0.86, 1.40) | 1.26  (1.09, 1.45) |  |  | - | - |  |
|  |  |  |  |  |  |  |  |

CI indicates confidence interval; INS, insomnia symptoms; SLD, sleep duration; and CT, chronotype.

* Adjustments were performed as in Model 2, Table 2.

Table S5: Hazard ratios (95% CIs)* for acute myocardial infarction according to insomnia symptoms, sleep duration, chronotype and their joint association stratified by shift work (or night shifts) in UK Biobank (UKBB) and HUNT2.

|  | **UK Biobank** | | |  | **HUNT2** | | |
| --- | --- | --- | --- | --- | --- | --- | --- |
|  | **Non-shift workers** | **Shift workers** | **P for interaction** |  | **Non-shift workers** | **Shift workers** | **P for interaction** |
| **Exposures** |  |  |  |  |  |  |  |
| Insomnia symptoms |  |  |  |  |  |  |  |
| No | Ref. | Ref. | 0.439 |  | Ref. | Ref. | 0.399 |
| Yes | 1.10  (1.04, 1.16) | 1.20  (0.95, 1.51) |  |  | 1.07  (0.96, 1.20) | 1.20  (0.88, 1.64) |  |
|  |  |  |  |  |  |  |  |
| Sleep duration |  |  |  |  |  |  |  |
| Short | 1.10  (1.04, 1.16) | 1.05  (0.84, 1.30) |  |  | 1.06  (0.88, 1.28) | 0.99  (0.67, 1.45) |  |
| Normal | Ref. | Ref. | 0.842 |  | Ref. | Ref. | 0.657 |
| Long | 1.14  (1.05, 1.24) | 1.05  (0.66, 1.66) |  |  | 0.98  (0.89, 1.08) | 0.86  (0.62, 1.19) |  |
|  |  |  |  |  |  |  |  |
| Chronotype |  |  |  |  |  |  |  |
| Morning | Ref. | Ref. | 0.519 |  | - | - |  |
| Evening | 1.08  (1.03, 1.14) | 1.00  (0.81, 1.24) |  |  | - | - |  |
|  |  |  |  |  |  |  |  |
| Insomnia symptoms (INS) and Sleep duration (SLD) |  |  |  |  |  |  |  |
| No INS & Short SLD | 1.07  (0.99, 1.15) | 1.12  (0.86, 1.45) |  |  | 0.99  (0.79, 1.25) | 1.13  (0.74, 1.73) |  |
| No INS & Normal SLD | Ref. | Ref. | 0.678 |  | Ref. | Ref. | 0.487 |
| No INS & Long SLD | 1.09  (0.99, 1.20) | 1.02  (0.60, 1.73) |  |  | 0.98  (0.88, 1.09) | 0.91  (0.65, 1.28) |  |
| INS & Short SLD | 1.16  (1.07, 1.25) | 1.11  (0.80, 1.53) |  |  | 1.27  (0.92, 1.74) | 0.72  (0.30, 1.76) |  |
| INS & Normal SLD | 1.06  (0.98, 1.14) | 1.39  (1.00, 1.92) |  |  | 1.05  (0.91, 1.22) | 1.39  (0.98, 1.98) |  |
| INS & Long SLD | 1.39  (1.20, 1.62) | 1.58  (0.64, 3.88) |  |  | 1.03  (0.85, 1.25) | 0.84  (0.34, 2.10) |  |
|  |  |  |  |  |  |  |  |
| Insomnia symptoms (INS) and Chronotype (CT) |  |  |  |  |  |  |  |
| No INS & Morning CT | Ref. | Ref. | 0.696 |  | - | - |  |
| No INS & Evening CT | 1.08  (1.02, 1.15) | 1.06  (0.83, 1.35) |  |  | - | - |  |
| INS & Morning CT | 1.10  (1.03, 1.18) | 1.32  (0.97, 1.80) |  |  | - | - |  |
| INS & Evening CT | 1.19  (1.10, 1.29) | 1.13  (0.80, 1.59) |  |  | - | - |  |
|  |  |  |  |  |  |  |  |
| Chronotype (CT) and Sleep duration (SLD) |  |  |  |  |  |  |  |
| Morning CT & Short SLD | 1.10  (1.02, 1.18) | 1.10  (0.83, 1.47) |  |  | - | - |  |
| Morning CT & Normal SLD | Ref. | Ref. | 0.825 |  | - | - |  |
| Morning CT & Long SLD | 1.14  (1.02, 1.27) | 1.30  (0.71, 2.37) |  |  | - | - |  |
| Evening CT & Short SLD | 1.19  (1.09, 1.30) | 1.05  (0.76, 1.45) |  |  | - | - |  |
| Evening CT & Normal SLD | 1.08  (1.02, 1.15) | 1.08  (0.82, 1.42) |  |  | - | - |  |
| Evening CT & Long SLD | 1.23  (1.09, 1.39) | 0.87  (0.42, 1.78) |  |  | - | - |  |
|  |  |  |  |  |  |  |  |

CI indicates confidence interval; INS, insomnia symptoms; SLD, sleep duration; and CT, chronotype.

* Adjustments were performed as in Model 2, Table 2.

Table S6: Hazard ratios (95% CIs)* for acute myocardial infarction according to insomnia symptoms, sleep duration, chronotype and their joint association stratified by depression in UK Biobank (UKBB) and HUNT2.

|  | **UK Biobank** | | |  | **HUNT2** | | |
| --- | --- | --- | --- | --- | --- | --- | --- |
|  | **Without depression** | **With depression** | **P for interaction** |  | **Without depression** | **With depression** | **P for interaction** |
| **Exposures** |  |  |  |  |  |  |  |
| Insomnia symptoms |  |  |  |  |  |  |  |
| No | Ref. | Ref. | 0.736 |  | Ref. | Ref. | 0.600 |
| Yes | 1.08  (1.02, 1.14) | 1.11  (0.98, 1.26) |  |  | 1.10  (0.97, 1.25) | 0.96  (0.72, 1.26) |  |
|  |  |  |  |  |  |  |  |
| Sleep duration |  |  |  |  |  |  |  |
| Short | 1.09  (1.03, 1.16) | 1.05  (0.91, 1.22) |  |  | 1.11  (0.92, 1.33) | 1.07  (0.67, 1.69) |  |
| Normal | Ref. | Ref. | 0.847 |  | Ref. | Ref. | 0.841 |
| Long | 1.09  (0.99, 1.20) | 1.13  (0.94, 1.35) |  |  | 0.93  (0.84, 1.05) | 0.97  (0.74, 1.26) |  |
|  |  |  |  |  |  |  |  |
| Chronotype |  |  |  |  |  |  |  |
| Morning | Ref. | Ref. | 0.997 |  | - | - |  |
| Evening | 1.06  (1.01, 1.12) | 1.06  (0.94, 1.21) |  |  | - | - |  |
|  |  |  |  |  |  |  |  |
| Insomnia symptoms (INS) and Sleep duration (SLD) |  |  |  |  |  |  |  |
| No INS & Short SLD | 1.09  (1.00, 1.17) | 1.01  (0.81, 1.26) |  |  | 1.10  (0.90, 1.36) | 0.87  (0.44, 1.73) |  |
| No INS & Normal SLD | Ref. | Ref. | 0.932 |  | Ref. | Ref. | 0.947 |
| No INS & Long SLD | 1.06  (0.96, 1.18) | 1.00  (0.80, 1.26) |  |  | 0.93  (0.84, 1.04) | 0.99  (0.72, 1.35) |  |
| INS & Short SLD | 1.13  (1.04, 1.23) | 1.10  (0.92, 1.31) |  |  | 1.18  (0.83, 1.68) | 1.23  (0.66, 2.30) |  |
| INS & Normal SLD | 1.06  (0.98, 1.14) | 1.04  (0.87, 1.25) |  |  | 1.11  (0.95, 1.29) | 0.94  (0.66, 1.35) |  |
| INS & Long SLD | 1.27  (1.05, 1.52) | 1.45  (1.10, 1.90) |  |  | 1.00  (0.80, 1.25) | 0.87  (0.56, 1.33) |  |
|  |  |  |  |  |  |  |  |
| Insomnia symptoms (INS) and Chronotype (CT) |  |  |  |  |  |  |  |
| No INS & Morning CT | Ref. | Ref. | 0.888 |  | - | - |  |
| No INS & Evening CT | 1.07  (1.00, 1.14) | 1.11  (0.94, 1.31) |  |  | - | - |  |
| INS & Morning CT | 1.08  (1.00, 1.16) | 1.16  (0.98, 1.38) |  |  | - | - |  |
| INS & Evening CT | 1.15  (1.05, 1.25) | 1.16  (0.97, 1.40) |  |  | - | - |  |
|  |  |  |  |  |  |  |  |
| Chronotype (CT) and Sleep duration (SLD) |  |  |  |  |  |  |  |
| Morning CT & Short SLD | 1.10  (1.02, 1.18) | 1.07  (0.88, 1.29) |  |  | - | - |  |
| Morning CT & Normal SLD | Ref. | Ref. | 0.991 |  | - | - |  |
| Morning CT & Long SLD | 1.12  (0.99, 1.26) | 1.11  (0.86, 1.45) |  |  | - | - |  |
| Evening CT & Short SLD | 1.17  (1.06, 1.29) | 1.11  (0.90, 1.37) |  |  | - | - |  |
| Evening CT & Normal SLD | 1.07  (1.01, 1.15) | 1.06  (0.90, 1.26) |  |  | - | - |  |
| Evening CT & Long SLD | 1.13  (0.98, 1.30) | 1.20  (0.94, 1.53) |  |  | - | - |  |
|  |  |  |  |  |  |  |  |

CI indicates confidence interval; INS, insomnia symptoms; SLD, sleep duration; and CT, chronotype.

* Adjustments were performed as in Model 3, Table 2.

Table S7: Hazard ratios (95% CIs)* for acute myocardial infarction according to insomnia symptoms, sleep duration, chronotype and their joint association stratified by anxiety in UK Biobank (UKBB) and HUNT2.

|  | **UK Biobank** | | |  | **HUNT2** | | |
| --- | --- | --- | --- | --- | --- | --- | --- |
|  | **Without anxiety** | **With anxiety** | **P for interaction** |  | **Without anxiety** | **With anxiety** | **P for interaction** |
| **Exposures** |  |  |  |  |  |  |  |
| Insomnia symptoms |  |  |  |  |  |  |  |
| No | Ref. | Ref. | 0.991 |  | Ref. | Ref. | 0.992 |
| Yes | 1.08  (1.02, 1.14) | 1.12  (0.95, 1.31) |  |  | 1.07  (0.94, 1.23) | 1.07  (0.85, 1.35) |  |
|  |  |  |  |  |  |  |  |
| Sleep duration |  |  |  |  |  |  |  |
| Short | 1.09  (1.03, 1.16) | 1.05  (0.88, 1.25) |  |  | 1.15  (0.95, 1.38) | 0.88  (0.58, 1.33) |  |
| Normal | Ref. | Ref. | 0.406 |  | Ref. | Ref. | 0.632 |
| Long | 1.11  (1.02, 1.21) | 1.01  (0.79, 1.29) |  |  | 0.94  (0.85, 1.04) | 0.90  (0.70, 1.15) |  |
|  |  |  |  |  |  |  |  |
| Chronotype |  |  |  |  |  |  |  |
| Morning | Ref. | Ref. | 0.881 |  | - | - |  |
| Evening | 1.06  (1.01, 1.12) | 1.08  (0.92, 1.26) |  |  | - | - |  |
|  |  |  |  |  |  |  |  |
| Insomnia symptoms (INS) and Sleep duration (SLD) |  |  |  |  |  |  |  |
| No INS & Short SLD | 1.09  (1.00, 1.17) | 0.97  (0.75, 1.27) |  |  | 1.12  (0.91, 1.38) | 0.71  (0.36, 1.41) |  |
| No INS & Normal SLD | Ref. | Ref. | 0.804 |  | Ref. | Ref. | 0.842 |
| No INS & Long SLD | 1.06  (0.96, 1.17) | 0.97  (0.72, 1.32) |  |  | 0.96  (0.86, 1.06) | 0.86  (0.62, 1.18) |  |
| INS & Short SLD | 1.12  (1.04, 1.22) | 1.13  (0.92, 1.41) |  |  | 1.29  (0.89, 1.87) | 1.02  (0.60, 1.73) |  |
| INS & Normal SLD | 1.05  (0.97, 1.13) | 1.07  (0.86, 1.33) |  |  | 1.09  (0.92, 1.29) | 1.01  (0.76, 1.35) |  |
| INS & Long SLD | 1.35  (1.15, 1.59) | 1.17  (0.78, 1.75) |  |  | 0.94  (0.74, 1.19) | 0.98  (0.68, 1.41) |  |
|  |  |  |  |  |  |  |  |
| Insomnia symptoms (INS) and Chronotype (CT) |  |  |  |  |  |  |  |
| No INS & Morning CT | Ref. | Ref. | 0.997 |  | - | - |  |
| No INS & Evening CT | 1.07  (1.01, 1.14) | 1.07  (0.88, 1.31) |  |  | - | - |  |
| INS & Morning CT | 1.09  (1.01, 1.17) | 1.11  (0.90, 1.37) |  |  | - | - |  |
| INS & Evening CT | 1.14  (1.04, 1.24) | 1.21  (0.96, 1.52) |  |  | - | - |  |
|  |  |  |  |  |  |  |  |
| Chronotype (CT) and Sleep duration (SLD) |  |  |  |  |  |  |  |
| Morning CT & Short SLD | 1.10  (1.02, 1.18) | 1.06  (0.85, 1.33) |  |  | - | - |  |
| Morning CT & Normal SLD | Ref. | Ref. | 0.381 |  | - | - |  |
| Morning CT & Long SLD | 1.10  (0.98, 1.23) | 1.22  (0.88, 1.69) |  |  | - | - |  |
| Evening CT & Short SLD | 1.16  (1.06, 1.27) | 1.19  (0.92, 1.54) |  |  | - | - |  |
| Evening CT & Normal SLD | 1.06  (1.00, 1.14) | 1.14  (0.93, 1.40) |  |  | - | - |  |
| Evening CT & Long SLD | 1.19  (1.04, 1.35) | 0.92  (0.64, 1.33) |  |  | - | - |  |
|  |  |  |  |  |  |  |  |

CI indicates confidence interval; INS, insomnia symptoms; SLD, sleep duration; and CT, chronotype.

* Adjustments were performed as in Model 3, Table 2.

Table S8: Hazard ratios (95% CIs) for acute myocardial infarction (AMI) according to self-reported insomnia, sleep duration and chronotype excluding the first two years of follow-up in UK Biobank (UKBB) and HUNT2.

|  |  | **Insomnia** | |  | **Sleep duration** | | |  | **Chronotype** | |
| --- | --- | --- | --- | --- | --- | --- | --- | --- | --- | --- |
|  |  | **No** | **Yes** |  | **Short** | **Normal** | **Long** |  | **Morning** | **Evening** |
|  |  |  |  |  |  |  |  |  |  |  |
| **UK Biobank**  (n = 300 385) | AMI events/  Person-years | 4 277/  2 581 934 | 1 812/  964 084 |  | 1 592/  843 405 | 3 906/  2 444 472 | 591/  258 141 |  | 3 734/  2 228 511 | 2 355/  1 317 507 |
|  | Model 1 | Ref. | 1.16  (1.10, 1.23) |  | 1.20  (1.13, 1.27) | Ref. | 1.24  (1.13, 1.35) |  | Ref. | 1.16  (1.10, 1.22) |
|  | Model 2 | Ref. | 1.09  (1.03, 1.16) |  | 1.09  (1.03, 1.16) | Ref. | 1.11  (1.02, 1.22) |  | Ref. | 1.10  (1.05, 1.16) |
|  | Model 3 | Ref. | 1.07  (1.01, 1.13) |  | 1.08  (1.02, 1.15) | Ref. | 1.07  (0.98, 1.17) |  | Ref. | 1.09  (1.03, 1.14) |
|  |  |  |  |  |  |  |  |  |  |  |
| **HUNT2**  (n = 30 464) | AMI events/  Person-years | 1 999/  576 661 | 391/  76 290 |  | 144/  41 265 | 1 595/  469 968 | 651/  141 718 |  | - | - |
|  | Model 1 | Ref. | 1.15  (1.03, 1.29) |  | 1.15  (0.97, 1.36) | Ref. | 0.98  (0.89, 1.08) |  | - | - |
|  | Model 2 | Ref. | 1.09  (0.97, 1.21) |  | 1.06  (0.89, 1.26) | Ref. | 0.92  (0.84, 1.02) |  | - | - |
|  | Model 3 | Ref. | 1.08  (0.95, 1.21) |  | 1.10  (0.93, 1.31) | Ref. | 0.90  (0.82, 0.99) |  | - | - |
|  |  |  |  |  |  |  |  |  |  |  |

**Model 1**, adjusted for age and gender.

**Model 2**, adjusted for covariates in Model 1, along with marital status, alcohol intake frequency, smoking status, body mass index, physical activity, education, Townsend deprivation index (for UKBB), ethnicity (for UKBB), shift work, and employment status.

**Model 3**, adjusted for covariates in Model 2, along with systolic blood pressure, serum cholesterol level, blood glucose level, time since last meal, use of sleep medication(s), depression, and anxiety.

Table S9: Hazard ratios (95% confidence intervals) for acute myocardial infarction (AMI) according to the joint association of self-reported insomnia symptoms and sleep duration excluding the first two years of follow-up in UK Biobank (UKBB) and HUNT2.

|  |  | **No insomnia symptoms** | | |  | **Insomnia symptoms** | | |
| --- | --- | --- | --- | --- | --- | --- | --- | --- |
|  |  | **Sleep duration** | | |  | **Sleep duration** | | |
|  |  | **Short** | **Normal** | **Long** |  | **Short** | **Normal** | **Long** |
|  |  |  |  |  |  |  |  |  |
| **UK Biobank**  (n = 300 385) | AMI events/  Person-years | 808/  427 384 | 3 033/  1 950 013 | 436/  204 537 |  | 784/  416 020 | 873/  494 459 | 155/  53 604 |
|  | Model 1 | 1.18  (1.09, 1.27) | Ref. | 1.19  (1.07, 1.31) |  | 1.29  (1.19, 1.39) | 1.12  (1.04, 1.20) | 1.57  (1.34, 1.85) |
|  | Model 2 | 1.08  (1.00, 1.17) | Ref. | 1.08  (0.98, 1.20) |  | 1.14  (1.05, 1.23) | 1.07  (0.99, 1.16) | 1.32  (1.12, 1.55) |
|  | Model 3 | 1.08  (1.00, 1.17) | Ref. | 1.04  (0.94, 1.16) |  | 1.10  (1.02, 1.20) | 1.05  (0.98, 1.14) | 1.23  (1.04, 1.45) |
|  |  |  |  |  |  |  |  |  |
| **HUNT2**  (n = 30 464) | AMI events/  Person-years | 103/  32 939 | 1 357/  420 657 | 539/  123 065 |  | 41/  8 326 | 238/  49 311 | 112/  18 653 |
|  | Model 1 | 1.12  (0.92, 1.37) | Ref. | 0.99  (0.90, 1.10) |  | 1.32  (0.97, 1.81) | 1.17  (1.02, 1.35) | 1.08  (0.89, 1.31) |
|  | Model 2 | 1.05  (0.86, 1.28) | Ref. | 0.93  (0.84, 1.04) |  | 1.15  (0.84, 1.57) | 1.10  (0.96, 1.27) | 0.96  (0.79, 1.17) |
|  | Model 3 | 1.11  (0.90, 1.35) | Ref. | 0.91  (0.82, 1.01) |  | 1.15  (0.84, 1.58) | 1.09  (0.95, 1.27) | 0.92  (0.75, 1.13) |
|  |  |  |  |  |  |  |  |  |

**Model 1**, adjusted for age and gender.

**Model 2**, adjusted for covariates in Model 1, along with marital status, alcohol intake frequency, smoking status, body mass index, physical activity, education, Townsend deprivation index (for UKBB), ethnicity (for UKBB), shift work, and employment status.

**Model 3**, adjusted for covariates in Model 2, along with systolic blood pressure, serum cholesterol level, blood glucose level, time since last meal, use of sleep medication(s), depression, and anxiety.

Table S10: Hazard ratios (95% confidence intervals) for acute myocardial infarction (AMI) according to the joint association of self-reported insomnia symptoms and chronotype excluding the first two years of follow-up in UK Biobank.

|  | | | | **No insomnia symptoms** | | | |  | | **Insomnia symptoms** | | | |
| --- | --- | --- | --- | --- | --- | --- | --- | --- | --- | --- | --- | --- | --- |
|  |  |  |  | **Chronotype** | | | |  | | **Chronotype** | | | |
|  |  |  |  | **Morning** | | **Evening** | |  | | **Morning** | | **Evening** | |
|  | |  | |  | |  | |  | |  | |  | |
| **UK Biobank**  (n = 300 385) | | AMI events/  Person-years | | 2 634/  1 624 446 | | 1 643/  957 489 | |  | | 1 100/  604 066 | | 712/  360 018 | |
|  |  | Model 1 | | Ref. | | 1.14  (1.07, 1.21) | |  | | 1.14  (1.06, 1.22) | | 1.37  (1.26, 1.49) | |
|  |  | Model 2 | | Ref. | | 1.10  (1.03, 1.17) | |  | | 1.09  (1.02, 1.17) | | 1.21  (1.11, 1.31) | |
|  |  | Model 3 | | Ref. | | 1.09  (1.02, 1.16) | |  | | 1.07  (1.00, 1.15) | | 1.16  (1.06, 1.26) | |
|  | |  | |  | |  | |  | |  | |  | |

**Model 1**, adjusted for age and gender.

**Model 2**, adjusted for covariates in Model 1, along with marital status, alcohol intake frequency, smoking status, body mass index, physical activity, education, Townsend deprivation index, ethnicity, shift work, and employment status.

**Model 3**, adjusted for covariates in Model 2, along with systolic blood pressure, serum cholesterol level, blood glucose level, time since last meal, use of sleep medication(s), depression, and anxiety.

Table S11: Hazard ratios (95% confidence intervals) for acute myocardial infarction (AMI) according to the joint association of self-reported chronotype and sleep duration excluding the first two years of follow-up in UK Biobank.

|  |  | **Morning chronotype** | | |  | **Evening chronotype** | | |
| --- | --- | --- | --- | --- | --- | --- | --- | --- |
|  |  | **Sleep duration** | | |  | **Sleep duration** | | |
|  |  | **Short** | **Normal** | **Long** |  | **Short** | **Normal** | **Long** |
|  |  |  |  |  |  |  |  |  |
| **UK Biobank**  (n = 300 385) | AMI events/  Person-years | 1 006/  539 027 | 2 398/  1 539 879 | 330/  149 605 |  | 586/  304 377 | 1 508/  904 594 | 261/  108 536 |
|  | Model 1 | 1.20  (1.11, 1.29) | Ref. | 1.23  (1.09, 1.38) |  | 1.41  (1.28, 1.54) | 1.15  (1.08, 1.23) | 1.41  (1.24, 1.60) |
|  | Model 2 | 1.09  (1.02, 1.18) | Ref. | 1.12  (1.00, 1.26) |  | 1.21  (1.10, 1.32) | 1.11  (1.04, 1.18) | 1.21  (1.06, 1.37) |
|  | Model 3 | 1.08  (1.01, 1.17) | Ref. | 1.09  (0.97, 1.23) |  | 1.18  (1.08, 1.30) | 1.09  (1.02, 1.17) | 1.13  (0.99, 1.29) |
|  |  |  |  |  |  |  |  |  |

**Model 1**, adjusted for age and gender.

**Model 2**, adjusted for covariates in Model 1, along with marital status, alcohol intake frequency, smoking status, body mass index, physical activity, education, Townsend deprivation index, ethnicity, shift work, and employment status.

**Model 3**, adjusted for covariates in Model 2, along with systolic blood pressure, serum cholesterol level, blood glucose level, time since last meal, use of sleep medication(s), depression, and anxiety.

Table S12: Hazard ratios (95% confidence intervals) for acute myocardial infarction (AMI) according to self-reported insomnia symptoms, sleep duration and chronotype after adjusting for chronic disorders within UK Biobank (UKBB) and HUNT2.

|  |  | **Insomnia symptoms** | |  | **Sleep duration** | | |  | **Chronotype** | |
| --- | --- | --- | --- | --- | --- | --- | --- | --- | --- | --- |
|  |  | **No** | **Yes** |  | **Short** | **Normal** | **Long** |  | **Morning** | **Evening** |
|  |  |  |  |  |  |  |  |  |  |  |
| **UK Biobank**  (n = 297 170) | AMI events/  Person-years | 4 702/  2 543 217 | 2 006/  943 592 |  | 1 758/  825 662 | 4 286/  2 407 383 | 664/  253 764 |  | 4 130/  2 193 113 | 2 578/  1 293 696 |
|  | Model 1 | Ref. | 1.19  (1.13, 1.25) |  | 1.21  (1.14, 1.28) | Ref. | 1.30  (1.20, 1.41) |  | Ref. | 1.14  (1.08, 1.19) |
|  | Model 2 | Ref. | 1.06  (1.01, 1.12) |  | 1.07  (1.01, 1.13) | Ref. | 1.11  (1.02, 1.21) |  | Ref. | 1.07  (1.02, 1.12) |
|  | Model 3 | Ref. | 1.04  (0.99, 1.10) |  | 1.07  (1.01, 1.13) | Ref. | 1.08  (0.99, 1.17) |  | Ref. | 1.05  (1.00, 1.11) |
|  |  |  |  |  |  |  |  |  |  |  |
| **HUNT2**  (n = 30 604) | AMI events/  Person-years | 2 087/  568 946 | 408/  74 696 |  | 146/  40 478 | 1 641/  463 792 | 708/  139 371 |  | - | - |
|  | Model 1 | Ref. | 1.16  (1.04, 1.29) |  | 1.13  (0.96, 1.34) | Ref. | 1.05  (0.96, 1.15) |  | - | - |
|  | Model 2 | Ref. | 1.04  (0.93, 1.16) |  | 1.03  (0.87, 1.22) | Ref. | 0.95  (0.87, 1.05) |  | - | - |
|  | Model 3 | Ref. | 1.04  (0.93, 1.18) |  | 1.08  (0.91, 1.28) | Ref. | 0.93  (0.84, 1.02) |  | - | - |
|  |  |  |  |  |  |  |  |  |  |  |

**Model 1**, adjusted for age and gender.

**Model 2**, adjusted for covariates in Model 1, along with marital status, alcohol intake frequency, smoking status, body mass index, physical activity, education, Townsend deprivation index (for UKBB), ethnicity (for UKBB), shift work, employment status, and chronic disorders.

**Model 3**, adjusted for covariates in Model 2, along with systolic blood pressure, serum cholesterol level, blood glucose level, time since last meal, use of sleep medication(s), depression, and anxiety.

Table S13: Hazard ratios (95% confidence intervals) for acute myocardial infarction (AMI) according to the joint association of self-reported insomnia symptoms and sleep duration after adjusting for chronic disorders within UK Biobank (UKBB) and HUNT2.

|  |  | **No insomnia symptoms** | | |  | **Insomnia symptoms** | | |
| --- | --- | --- | --- | --- | --- | --- | --- | --- |
|  |  | **Sleep duration** | | |  | **Sleep duration** | | |
|  |  | **Short** | **Normal** | **Long** |  | **Short** | **Normal** | **Long** |
|  |  |  |  |  |  |  |  |  |
| **UK Biobank**  (n = 297 170) | AMI events/  Person-years | 883/  419 868 | 3 338/  1 922 214 | 481/  201 134 |  | 875/  405 794 | 948/  485 168 | 183/  52 629 |
|  | Model 1 | 1.16  (1.08, 1.25) | Ref. | 1.22  (1.11, 1.35) |  | 1.33  (1.23, 1.43) | 1.12  (1.04, 1.20) | 1.74  (1.50, 2.02) |
|  | Model 2 | 1.06  (0.98, 1.14) | Ref. | 1.06  (0.96, 1.17) |  | 1.10  (1.02, 1.19) | 1.03  (0.95, 1.10) | 1.31  (1.13, 1.52) |
|  | Model 3 | 1.07  (0.99, 1.15) | Ref. | 1.03  (0.93, 1.14) |  | 1.08  (1.00, 1.17) | 1.02  (0.95, 1.09) | 1.25  (1.07, 1.45) |
|  |  |  |  |  |  |  |  |  |
| **HUNT2**  (n = 30 604) | AMI events/  Person-years | 104/  32 417 | 1 399/  415 376 | 584/  121 153 |  | 42/  8 061 | 242/  48 416 | 124/  18 219 |
|  | Model 1 | 1.10  (0.90, 1.34) | Ref. | 1.06  (0.96, 1.17) |  | 1.33  (0.98, 1.80) | 1.17  (1.02, 1.34) | 1.18  (0.98, 1.42) |
|  | Model 2 | 1.02  (0.84, 1.25) | Ref. | 0.96  (0.87, 1.06) |  | 1.07  (0.79, 1.46) | 1.05  (0.92, 1.21) | 0.97  (0.80, 1.17) |
|  | Model 3 | 1.08  (0.89, 1.32) | Ref. | 0.93  (0.84, 1.03) |  | 1.10  (0.81, 1.50) | 1.06  (0.91, 1.22) | 0.93  (0.77, 1.14) |
|  |  |  |  |  |  |  |  |  |

**Model 1**, adjusted for age and gender.

**Model 2**, adjusted for covariates in Model 1, along with marital status, alcohol intake frequency, smoking status, body mass index, physical activity, education, Townsend deprivation index (for UKBB), ethnicity (for UKBB), shift work, employment status, and chronic disorders.

**Model 3**, adjusted for covariates in Model 2, along with systolic blood pressure, serum cholesterol level, blood glucose level, time since last meal, use of sleep medication(s), depression, and anxiety.

Table S14: Hazard ratios (95% confidence intervals) for acute myocardial infarction (AMI) according to the joint association of self-reported insomnia symptoms and chronotype after adjusting for chronic disorders within UK Biobank.

|  | | | | **No insomnia symptoms** | | | |  | | **Insomnia symptoms** | | | |
| --- | --- | --- | --- | --- | --- | --- | --- | --- | --- | --- | --- | --- | --- |
|  |  |  |  | **Chronotype** | | | |  | | **Chronotype** | | | |
|  |  |  |  | **Morning** | | **Evening** | |  | | **Morning** | | **Evening** | |
|  | |  | |  | |  | |  | |  | |  | |
| **UK Biobank**  (n = 297 170) | | AMI events/  Person-years | | 2 907/  1 601 216 | | 1 795/  942 000 | |  | | 1 223/  591 896 | | 783/  351 695 | |
|  |  | Model 1 | | Ref. | | 1.12  (1.06, 1.19) | |  | | 1.17  (1.09, 1.25) | | 1.37  (1.27, 1.49) | |
|  |  | Model 2 | | Ref. | | 1.07  (1.01, 1.13) | |  | | 1.06  (0.99, 1.14) | | 1.13  (1.04, 1.23) | |
|  |  | Model 3 | | Ref. | | 1.06  (1.00, 1.12) | |  | | 1.05  (0.98, 1.12) | | 1.10  (1.01, 1.19) | |
|  | |  | |  | |  | |  | |  | |  | |

**Model 1**, adjusted for age and gender.

**Model 2**, adjusted for covariates in Model 1, along with marital status, alcohol intake frequency, smoking status, body mass index, physical activity, education, Townsend deprivation index, ethnicity, shift work, employment status, and chronic disorders.

**Model 3**, adjusted for covariates in Model 2, along with systolic blood pressure, serum cholesterol level, blood glucose level, time since last meal, use of sleep medication(s), depression, and anxiety.

Table S15: Hazard ratios (95% confidence intervals) for acute myocardial infarction (AMI) according to the joint association of self-reported chronotype and sleep duration after adjusting for chronic disorders within UK Biobank.

|  |  | **Morning chronotype** | | |  | **Evening chronotype** | | |
| --- | --- | --- | --- | --- | --- | --- | --- | --- |
|  |  | **Sleep duration** | | |  | **Sleep duration** | | |
|  |  | **Short** | **Normal** | **Long** |  | **Short** | **Normal** | **Long** |
|  |  |  |  |  |  |  |  |  |
| **UK Biobank**  (n = 297 170) | AMI events/  Person-years | 1 124/  528 105 | 2 635/  1 517 813 | 371/  147 195 |  | 634/  297 557 | 1 651/  889 570 | 293/  106 569 |
|  | Model 1 | 1.22  (1.14, 1.31) | Ref. | 1.29  (1.16, 1.44) |  | 1.37  (1.25, 1.49) | 1.14  (1.07, 1.21) | 1.47  (1.30, 1.66) |
|  | Model 2 | 1.09  (1.02, 1.17) | Ref. | 1.12  (1.01, 1.25) |  | 1.13  (1.04, 1.24) | 1.08  (1.02, 1.15) | 1.17  (1.03, 1.32) |
|  | Model 3 | 1.08  (1.01, 1.16) | Ref. | 1.10  (0.99, 1.23) |  | 1.12  (1.03, 1.22) | 1.07  (1.01, 1.14) | 1.11  (0.98, 1.26) |
|  |  |  |  |  |  |  |  |  |

**Model 1**, adjusted for age and gender.

**Model 2**, adjusted for covariates in Model 1, along with marital status, alcohol intake frequency, smoking status, body mass index, physical activity, education, Townsend deprivation index, ethnicity, shift work, employment status, and chronic disorders.

**Model 3**, adjusted for covariates in Model 2, along with systolic blood pressure, serum cholesterol level, blood glucose level, time since last meal, use of sleep medication(s), depression, and anxiety.

Table S16: Hazard ratios (95% confidence intervals) for acute myocardial infarction (AMI) according to self-reported insomnia symptoms, sleep duration and chronotype within White British population in UK Biobank.

|  |  | **Insomnia symptoms** | |  | **Sleep duration** | | |  | **Chronotype** | |
| --- | --- | --- | --- | --- | --- | --- | --- | --- | --- | --- |
|  |  | **No** | **Yes** |  | **Short** | **Normal** | **Long** |  | **Morning** | **Evening** |
|  |  |  |  |  |  |  |  |  |  |  |
| **UK Biobank**  (n = 269 375) | AMI events/  Person-years | 4 290/  2 292 585 | 1 866/  872 425 |  | 1 585/  733 803 | 3 974/  2 200 379 | 597/  230 829 |  | 3 802/  1 999 431 | 2 354/  1 165 579 |
|  | Model 1 | Ref. | 1.19  (1.13, 1.26) |  | 1.20  (1.13, 1.27) | Ref. | 1.27  (1.16, 1.38) |  | Ref. | 1.14  (1.08, 1.20) |
|  | Model 2 | Ref. | 1.11  (1.05, 1.17) |  | 1.09  (1.03, 1.15) | Ref. | 1.12  (1.02, 1.22) |  | Ref. | 1.07  (1.02, 1.13) |
|  | Model 3 | Ref. | 1.09  (1.03, 1.15) |  | 1.08  (1.02, 1.15) | Ref. | 1.08  (0.99, 1.18) |  | Ref. | 1.06  (1.01, 1.12) |
|  |  |  |  |  |  |  |  |  |  |  |

**Model 1**, adjusted for age and gender.

**Model 2**, adjusted for covariates in Model 1, along with marital status, alcohol intake frequency, smoking status, body mass index, physical activity, education, Townsend deprivation index, shift work, and employment status.

**Model 3**, adjusted for covariates in Model 2, along with systolic blood pressure, serum cholesterol level, blood glucose level, time since last meal, use of sleep medication(s), depression, and anxiety.

Table S17: Hazard ratios (95% confidence intervals) for acute myocardial infarction (AMI) according to the joint association of self-reported insomnia and sleep duration within White British population in UK Biobank.

|  |  | **No insomnia symptoms** | | |  | **Insomnia symptoms** | | |
| --- | --- | --- | --- | --- | --- | --- | --- | --- |
|  |  | **Sleep duration** | | |  | **Sleep duration** | | |
|  |  | **Short** | **Normal** | **Long** |  | **Short** | **Normal** | **Long** |
|  |  |  |  |  |  |  |  |  |
| **UK Biobank**  (n = 269 375) | AMI events/  Person-years | 765/  361 048 | 3 089/  1 749 267 | 436/  182 270 |  | 820/  372 754 | 885/  451 111 | 161/  48 559 |
|  | Model 1 | 1.13  (1.05, 1.23) | Ref. | 1.21  (1.09, 1.34) |  | 1.33  (1.23, 1.44) | 1.11  (1.03, 1.20) | 1.64  (1.40, 1.92) |
|  | Model 2 | 1.04  (0.96, 1.13) | Ref. | 1.08  (0.97, 1.19) |  | 1.16  (1.08, 1.26) | 1.06  (0.98, 1.14) | 1.34  (1.14, 1.57) |
|  | Model 3 | 1.05  (0.97, 1.13) | Ref. | 1.05  (0.94, 1.16) |  | 1.14  (1.05, 1.23) | 1.05  (0.97, 1.13) | 1.27  (1.09, 1.50) |
|  |  |  |  |  |  |  |  |  |

**Model 1**, adjusted for age and gender.

**Model 2**, adjusted for covariates in Model 1, along with marital status, alcohol intake frequency, smoking status, body mass index, physical activity, education, Townsend deprivation index, shift work, and employment status.

**Model 3**, adjusted for covariates in Model 2, along with systolic blood pressure, serum cholesterol level, blood glucose level, time since last meal, use of sleep medication(s), depression, and anxiety.

Table S18: Hazard ratios (95% confidence intervals) for acute myocardial infarction (AMI) according to the joint association of self-reported insomnia symptoms and chronotype within White British population in UK Biobank.

|  | | | | **No insomnia symptoms** | | | |  | | **Insomnia symptoms** | | | |
| --- | --- | --- | --- | --- | --- | --- | --- | --- | --- | --- | --- | --- | --- |
|  |  |  |  | **Chronotype** | | | |  | | **Chronotype** | | | |
|  |  |  |  | **Morning** | | **Evening** | |  | | **Morning** | | **Evening** | |
|  | |  | |  | |  | |  | |  | |  | |
| **UK Biobank**  (n = 269 375) | | AMI events/  Person-years | | 2 652/  1 448 671 | | 1 638/  843 914 | |  | | 1 150/  550 760 | | 716/  321 666 | |
|  |  | Model 1 | | Ref. | | 1.13  (1.06, 1.20) | |  | | 1.18  (1.10, 1.26) | | 1.37  (1.26, 1.49) | |
|  |  | Model 2 | | Ref. | | 1.08  (1.01, 1.15) | |  | | 1.11  (1.04, 1.19) | | 1.18  (1.09, 1.29) | |
|  |  | Model 3 | | Ref. | | 1.07  (1.00, 1.14) | |  | | 1.09  (1.02, 1.17) | | 1.15  (1.06, 1.25) | |
|  | |  | |  | |  | |  | |  | |  | |

**Model 1**, adjusted for age and gender.

**Model 2**, adjusted for covariates in Model 1, along with marital status, alcohol intake frequency, smoking status, body mass index, physical activity, education, Townsend deprivation index, shift work, and employment status.

**Model 3**, adjusted for covariates in Model 2, along with systolic blood pressure, serum cholesterol level, blood glucose level, time since last meal, use of sleep medication(s), depression, and anxiety.

Table S19: Hazard ratios (95% confidence intervals) for acute myocardial infarction (AMI) according to the joint association of self-reported chronotype and sleep duration within White British population in UK Biobank.

|  |  | **Morning chronotype** | | |  | **Evening chronotype** | | |
| --- | --- | --- | --- | --- | --- | --- | --- | --- |
|  |  | **Sleep duration** | | |  | **Sleep duration** | | |
|  |  | **Short** | **Normal** | **Long** |  | **Short** | **Normal** | **Long** |
|  |  |  |  |  |  |  |  |  |
| **UK Biobank**  (n = 269 375) | AMI events/  Person-years | 1 008/  472 561 | 2 468/  1 392 551 | 326/  134 319 |  | 577/  261 242 | 1 506/  807 827 | 271/  96 510 |
|  | Model 1 | 1.19  (1.11, 1.28) | Ref. | 1.22  (1.09, 1.37) |  | 1.38  (1.26, 1.51) | 1.12  (1.05, 1.19) | 1.47  (1.29, 1.66) |
|  | Model 2 | 1.09  (1.01, 1.17) | Ref. | 1.10  (0.97, 1.23) |  | 1.17  (1.07, 1.28) | 1.07  (1.00, 1.14) | 1.21  (1.07, 1.38) |
|  | Model 3 | 1.08  (1.00, 1.16) | Ref. | 1.07  (0.95, 1.20) |  | 1.16  (1.05, 1.27) | 1.06  (0.99, 1.13) | 1.16  (1.02, 1.31) |
|  |  |  |  |  |  |  |  |  |

**Model 1**, adjusted for age and gender.

**Model 2**, adjusted for covariates in Model 1, along with marital status, alcohol intake frequency, smoking status, body mass index, physical activity, education, Townsend deprivation index, shift work, and employment status.

**Model 3**, adjusted for covariates in Model 2, along with systolic blood pressure, serum cholesterol level, blood glucose level, time since last meal, use of sleep medication(s), depression, and anxiety.

Table S20: Hazard ratios (95% confidence intervals) for acute myocardial infarction (AMI) according to self-reported insomnia symptoms and sleep duration in HUNT2 restricting the end of follow-up until December 31, 2008.

|  |  | **Insomnia symptoms** | |  | **Sleep duration** | | |
| --- | --- | --- | --- | --- | --- | --- | --- |
|  |  | **No** | **Yes** |  | **Short** | **Normal** | **Long** |
|  |  |  |  |  |  |  |  |
| **HUNT2**  (n = 31 091) | AMI events/  Person-years | 939/  317 050 | 205/  43 809 |  | 62/  22 521 | 691/  256 386 | 391/  81 952 |
|  | Model 1 | Ref. | 1.16  (0.99, 1.35) |  | 1.19  (0.92, 1.54) | Ref. | 1.03  (0.91, 1.18) |
|  | Model 2 | Ref. | 1.08  (0.93, 1.26) |  | 1.06  (0.82, 1.38) | Ref. | 0.99  (0.87, 1.13) |
|  | Model 3 | Ref. | 1.12  (0.94, 1.32) |  | 1.13  (0.87, 1.47) | Ref. | 0.97  (0.85, 1.11) |
|  |  |  |  |  |  |  |  |

**Model 1**, adjusted for age and gender.

**Model 2**, adjusted for covariates in Model 1, along with marital status, alcohol intake frequency, smoking status, body mass index, physical activity, education, shift work, and employment status.

**Model 3**, adjusted for covariates in Model 2, along with systolic blood pressure, serum cholesterol level, blood glucose level, time since last meal, use of sleep medication(s), depression, and anxiety.

Table S21: Hazard ratios (95% confidence intervals) for acute myocardial infarction (AMI) according to the joint association of self-reported insomnia symptoms and sleep duration in HUNT2 restricting the end of follow-up until December 31, 2008.

|  |  | **No insomnia symptoms** | | |  | **Insomnia symptoms** | | |
| --- | --- | --- | --- | --- | --- | --- | --- | --- |
|  |  | **Sleep duration** | | |  | **Sleep duration** | | |
|  |  | **Short** | **Normal** | **Long** |  | **Short** | **Normal** | **Long** |
|  |  |  |  |  |  |  |  |  |
| **HUNT2**  (n = 31 091) | AMI events/  Person-years | 43/  17 840 | 581/  228 578 | 315/  70 632 |  | 19/  4 681 | 110/  27 808 | 76/  11 320 |
|  | Model 1 | 1.17  (0.86, 1.59) | Ref. | 1.03  (0.89, 1.18) |  | 1.32  (0.84, 2.09) | 1.14  (0.93, 1.40) | 1.20  (0.94, 1.54) |
|  | Model 2 | 1.06  (0.78, 1.45) | Ref. | 0.99  (0.85, 1.14) |  | 1.09  (0.69, 1.73) | 1.08  (0.88, 1.32) | 1.08  (0.84, 1.39) |
|  | Model 3 | 1.14  (0.84, 1.56) | Ref. | 0.97  (0.84, 1.12) |  | 1.18  (0.74, 1.87) | 1.12  (0.90, 1.38) | 1.09  (0.83, 1.42) |
|  |  |  |  |  |  |  |  |  |

**Model 1**, adjusted for age and gender.

**Model 2**, adjusted for covariates in Model 1, along with marital status, alcohol intake frequency, smoking status, body mass index, physical activity, education, shift work, and employment status.

**Model 3**, adjusted for covariates in Model 2, along with systolic blood pressure, serum cholesterol level, blood glucose level, time since last meal, use of sleep medication(s), depression, and anxiety.

Table S22: List of medications used to define the sleep medications covariate in UKBB.

| **Sleep medication** | **Treatment/medication code**  (UKBB field ID: 20003) | |
| --- | --- | --- |
| Oxazepam | | 1140863442 |
| Meprobamate | | 1140863378 |
| Medazepam | | 1140863372 |
| Bromazepam | | 1140863318 |
| Lorazepam | | 1140863302 |
| Clobazam | | 1140863268 |
| Chlormezanone | | 1140863262, 1140868274 |
| Temazepam | | 1140863202 |
| Nitrazepam | | 1140863182, 1140863104 |
| Lormetazepam | | 1140863176 |
| Diazepam | | 1140863152, 1141157496 |
| Zopiclone | | 1140863144 |
| Triclofos sodium | | 1140863140 |
| Methyprylone | | 1140856040 |
| Prazepam | | 1140855944 |
| Triazolam | | 1140855914 |
| Ketazolam | | 1140855860 |
| Dichloralphenazone | | 1140855824 |
| Clomethiazole | | 1140909798 |
| Zaleplon | | 1141171404 |
| Butobarbital | | 1141180444 |
| Clonazepam | | 1140872150 |
| Flurazepam | | 1140863110 |
| Loprazolam | | 1140863120 |
| Alprazolam | | 1140863308 |
| Butobarbitone | | 1140882090 |
